# Anti-phospholipid antibodies as a risk factor for renal injury in patients with systemic lupus erythematosus: a comprehensive analysis

**DOI:** 10.3389/fimmu.2026.1734274

**Published:** 2026-02-03

**Authors:** Hui Guan, Chengzi Tian, Lefeng Chen, Wenjing Wang, Lihuan Zhang, Mingcheng Huang, Xiaofei Wang, Duo Chen

**Affiliations:** 1 Department of Radiation Oncology, The First Affiliated Hospital of Zhengzhou University, Zhengzhou, Henan, China; 2 Department of Gynecology, The First People’s Hospital of Yunnan Province, The Affiliated Hospital of Kunming University of Science and Technology, Kunming, Yunnan, China; 3 Department of Rheumatology, Sun Yat-sen Memorial Hospital of Sun Yat-sen University, Guangzhou, Guangdong, China; 4 Department of Biochemistry, SUSTech Homeostatic Medicine Institute, School of Medicine, Southern University of Science and Technology, Shenzhen, Guangdong, China; 5 Center for Translational Medicine, The First Affiliated Hospital of Zhengzhou University, Zhengzhou, Henan, China; 6 Department of Nephrology, Center of Kidney and Urology, The Seventh Affiliated Hospital, Sun Yat-sen University, Shenzhen, Guangdong, China; 7 Department of Endocrinology and Metabolism, The First Affiliated Hospital of Zhengzhou University, Zhengzhou, Henan, China

**Keywords:** anti-phospholipid antibodies, anti-phospholipid syndrome, lupus anticoagulant, renal injury, systemic lupus erythematosus

## Abstract

**Background:**

Although the existence of antiphospholipid antibodies (aPL) has been extensively documented as a risk factor for thrombocytopenia, hemolytic anemia, and recurrent miscarriage, their contribution to renal damage in the context of the systemic lupus erythematosus (SLE) is yet to be defined. This meta-analysis investigated the association between aPL and renal injury among patients with SLE.

**Methods:**

A systematic literature search was conducted to determine publications that examined the relationship between the level of aPL and renal functioning in SLE patients in four electronic databases (PubMed, Cochrane Library, Embase, and Web of Science). Funnel plots and Egger’s test were utilized to assess the presence of publication bias. Sensitivity analysis and the trim-and-fill method were used in the evaluation of the stability of the results. Subgroup analyses were performed according to study design, geographic region, aPL subtype, publication date, and pathological type of lupus nephritis. Also, the cumulative meta-analyses were conducted by ranking the studies based on the year of publication, sample size, and the Newcastle-Ottawa Scale score.

**Results:**

A total of 34,353 publications were retrieved up to September 12, 2025. After screening, a total of 70 studies (18 case-control, 23 cohort, and 29 cross-sectional) involving 12,456 SLE patients were included. The pooled OR for renal injury in aPL−positive versus aPL−negative patients was 2.09 (1.70–2.58). Subgroup analysis revealed anti-cardiolipin (aCL), lupus anticoagulant (LA), and antiphospholipid syndrome significantly increased the risk of renal injury compared with control groups, 108with OR of 1.71 (1.34–2.18), 2.43 (1.64–3.61), 2.07 (1.48–2.89), respectively. In contrast, no statistically significant increase in renal injury risk was observed in groups positive for anti-β2-glycoprotein I and aPS/PT. Cumulative meta-analyses consistently demonstrated an increased risk of renal injury in aPL-positive patients, and this association remained stable across different publication years, sample sizes, and study quality.

**Conclusions:**

Seropositivity for aPL was significantly associated with an increased risk of renal injury in SLE patients, primarily driven by LA and aCL.

## Introduction

Systemic lupus erythematosus (SLE) is a systemic, autoimmune, inflammatory disease that involves multiple organs and predominantly occurs in reproductive-aged women, with a female-to-male ratio of approximately 9:1 (1). The incidence of SLE varies substantially across regions, with estimates ranging from 3.7 to 49.0 per 100,000 person-years in North America, 1.5 to 7.4 in Europe, 1.4 to 6.3 in South America, and 2.8 to 8.6 in Asia (2). Evidence suggests that genetic predisposition, ultraviolet exposure, high estrogen levels, and infections are implicated in the pathogenesis of SLE. It has been reported that nearly 40% of SLE patients progress to lupus nephritis with clinical manifestations such as hematuria, pyuria, proteinuria, and hypoalbuminemia, and 10%-20% of patients eventually develop end-stage renal disease or die (3). The mechanism of renal injury in SLE patients remains unclear and involves various factors such as genetics, environment, and drugs. Exposure to autoantigens activates B cells to produce large amounts of autoantibodies, and antibody deposition in the kidney is an important cause of renal injury (4, 5).

Anti-phospholipid antibodies (aPL) are autoantibodies targeting an array of negatively charged phospholipids, phospholipid-binding proteins, and their complexes (6). The primary aPL profiles utilized in clinical practice encompass anti-β2-glycoprotein I (aβ2GPI) IgG/IgM antibodies, anti-cardiolipin (aCL) IgG/IgM antibodies, and lupus anticoagulant (LA) (7). The aPL are shown to be related to thrombocytopenia, hemolytic anemia, arteriovenous thrombosis, neuropsychiatric symptoms, and recurrent miscarriages in SLE patients (8–10). In addition to the conventional aPL, SLE patients possess a broad spectrum of non-criteria aPL, such as anti-phosphatidylserine antibodies (aPS), anti-prothrombin antibodies (aPT), and their complex-targeting antibodies (aPS/PT), which also play significant roles in organ damage. Several studies have confirmed that autoantibodies secreted by B cells-not only IgG and IgM but also IgA isotype modulate the progression of SLE through distinct mechanisms (11).

However, a clear consensus is lacking regarding the precise contribution of aPL to SLE-associated renal injury. Some studies have shown a correlation between aPL and renal thrombotic microangiopathy, renal infarction, and chronic renal insufficiency, whereas others have not observed any significant associations (12, 13). The aPL mainly exert their prothrombotic effects by binding to or cross-linking various hemostatic and fibrinolytic proteins (such as plasmin, thrombin), as well as coagulation factor X, thereby disrupting the dynamic balance between coagulation and fibrinolysis and promoting thrombosis. The resulting thrombotic events lead to chronic ischemic and hypoxic injury of nephrons and ultimately impair renal function (14). By binding to phospholipids on the cell membrane, aPL activate endothelial cells, upregulate adhesion molecules, recruit infiltrating neutrophils and macrophages, and stimulate the release of pro−inflammatory cytokines, thus triggering local inflammation (15). In addition, aPL can disrupt the glomerular charge barrier, enhance oxidative stress in podocytes, and promote foot process effacement, thereby damaging the glomerular filtration barrier (16). These mechanisms collectively contribute to renal injury in SLE. In the present study, we systematically retrieved all relevant clinical studies and performed a meta-analysis to quantitatively investigate the association between aPL and the risk of renal injury in SLE.

## Methods

### Meta-analysis protocol

This meta-analysis was registered prospectively in the PROSPERO database (CRD420251145946, https://www.crd.york.ac.uk/PROSPERO). The study was designed following PRISMA guidelines (**Supplementary Table S1**) (17).

### Literature retrieval and screening

A comprehensive literature search was undertaken in PubMed, Embase, Cochrane Library, and Web of Science up to September 12, 2025. Search terms included “systemic lupus erythematosus”, “lupus nephritis”, “anti-phospholipid”, “anti-cardiolipin”, “anti-β2-glycoprotein I”, “lupus anticoagulant”, “anti-phosphatidylserine”, “anti-prothrombin”, “anti-phosphatidylserine/prothrombin”, “anti-phosphatidic acid”, “anti-phosphatidylinositol”, “anti-phosphatidylcholine”, “anti-phosphatidylethanolamine”, “anti-protein C”, “anti-protein S”, “anti-annexin A2”, and “anti-annexin A5”. There were no restrictions on study design, date of publication, or language. The specific search strategies were provided in **Supplementary Table S2**. In addition, manual searches were performed for potentially relevant studies. The literature search and selection were performed independently by two investigators, with any disagreements adjudicated by a third investigator.

### Inclusion and exclusion criteria

The inclusion criteria for the studies were as follows (1): the population studied was diagnosed as SLE patients (2); the proportion of patients with renal injury was reported (3); the positivity status and type of aPL were reported (4). The acceptable study designs included cross-sectional, cohort studies and case-control studies.

The exclusion criteria were as follows (1): letters, reviews, case reports, and meeting abstracts; (2) duplicate studies; (3) unavailability of the full text; (4) studies that did not give a control group; (5) studies that did not provide sufficient data to estimate the association between aPL and renal injury.

### Data extraction

Data, including the title, first author, publication year, study type, sample size, whether the study was multicenter, age of patients, gender ratio of patients, inclusion period, country, type and positivity percentages of reported aPL and renal outcome were extracted. For studies that reported multiple aPL assays, data on the association between all available aPL types and renal injury were extracted. We established a predefined priority order for studies reporting multiple aPL subtypes in the pooled analysis, based on Domingues et al. and the relative clinical significance of these antibodies (18). Because “any aPL positivity” reflects the overall burden of aPL subtypes and offers higher sensitivity, it was assigned the highest priority. Among individual antibodies, aCL is the most frequently detected aPL subtype and therefore ranked above aβ2GPI, LA, and anti-phospholipid syndrome (APS). Both aCL and aβ2GPI include three isotypes: IgG, IgM, and IgA. IgG is the most commonly implicated pathogenic isotype, followed by IgM, whereas IgA has a lower detection rate and its clinical relevance remains uncertain. Accordingly, the priority order was defined as follows: aCL IgG > aCL IgM > aCL IgA, and aβ2GPI IgG > aβ2GPI IgM > aβ2GPI IgA. All data were extracted independently by two investigators and disagreements were resolved with a third investigator.

### Quality assessment of included studies

The methodological quality of eligible studies was assessed using the Newcastle-Ottawa Scale (NOS) (19). The NOS evaluates studies across three domains: Selection (4 stars), Comparability (2 stars), and either Exposure (for case-control studies) or outcome (for cohort studies) (3 stars). For cross-sectional studies, a modified version of the NOS was applied, which employs the domains of sample selection (2 stars), assessment of exposure and outcome (4 stars), and confounding control (3 stars) (20). The total score ranges from 0 to 9 stars, with studies categorized as low (0-3), moderate (4-6), or high (7-9) quality.

### Sensitivity analysis

Sensitivity analysis was performed by sequentially omitting one study at a time to assess the robustness of the pooled estimates. The effect of the following criteria on the final renal outcome was also examined: (1) the study design (cohort, cross-sectional, case-control); (2) location (Africa, Americas, Asia, Europe); (3) publication period (1980-1989, 1990-1999, 2000-2009, 2010-2019, 2020-2025); (4) type of aPL; and (5) pathological class of lupus nephritis (I–VI).

### Heterogeneity assessment

Heterogeneity across studies was assessed using the I² statistic. An I^2^ value of not more than 50% was considered as low heterogeneity, and a fixed-effects model was applied; otherwise, a random-effects model was used.

### Publication bias

Some non-significant results are less likely to be published, leading to publication bias. We have evaluated the risk of publication bias by checking the symmetry of the funnel plot and use of Egger’s test. Lastly, the trim-and-fill method was used to assess the possible effects of the publication bias on the meta-analysis outcomes. This method estimates the number of missing studies in an asymmetric funnel plot and assesses the change in the pooled effect size before and after imputing these studies.

### Cumulative meta-analysis

Cumulative meta-analysis was applied to assessed the trend of risk of kidney injury between both groups by increasing the number of studies one by one according to publication time or study size or NOS score.

### Statistical analysis

All of the statistical analyses were conducted using Review Manager (RevMan version 5.3 Cochrane Collaboration, UK) and Stata (version 15.1.1, Stata Corporation, USA). In RevMan, we generated forest plots, performed tests of heterogeneity, and conducted subgroup analyses. The association between aPL and renal injury was expressed as odds ratios (OR) with 95% confidence intervals (CI). An OR > 1 indicated a higher risk of renal injury in the aPL-positive group, an OR < 1 indicated a lower risk, and an OR = 1 indicated no difference between groups. We constructed the funnel plot, Egger’s test, and trim and fill method to assess potential publication bias using Stata. The sensitivity analysis was performed as well by sequentially removing individual studies so as to investigate the stability of the pooled results. A two-sided P value < 0.05 was considered statistically significant.

## Results

### Literature search

**Figure 1:**
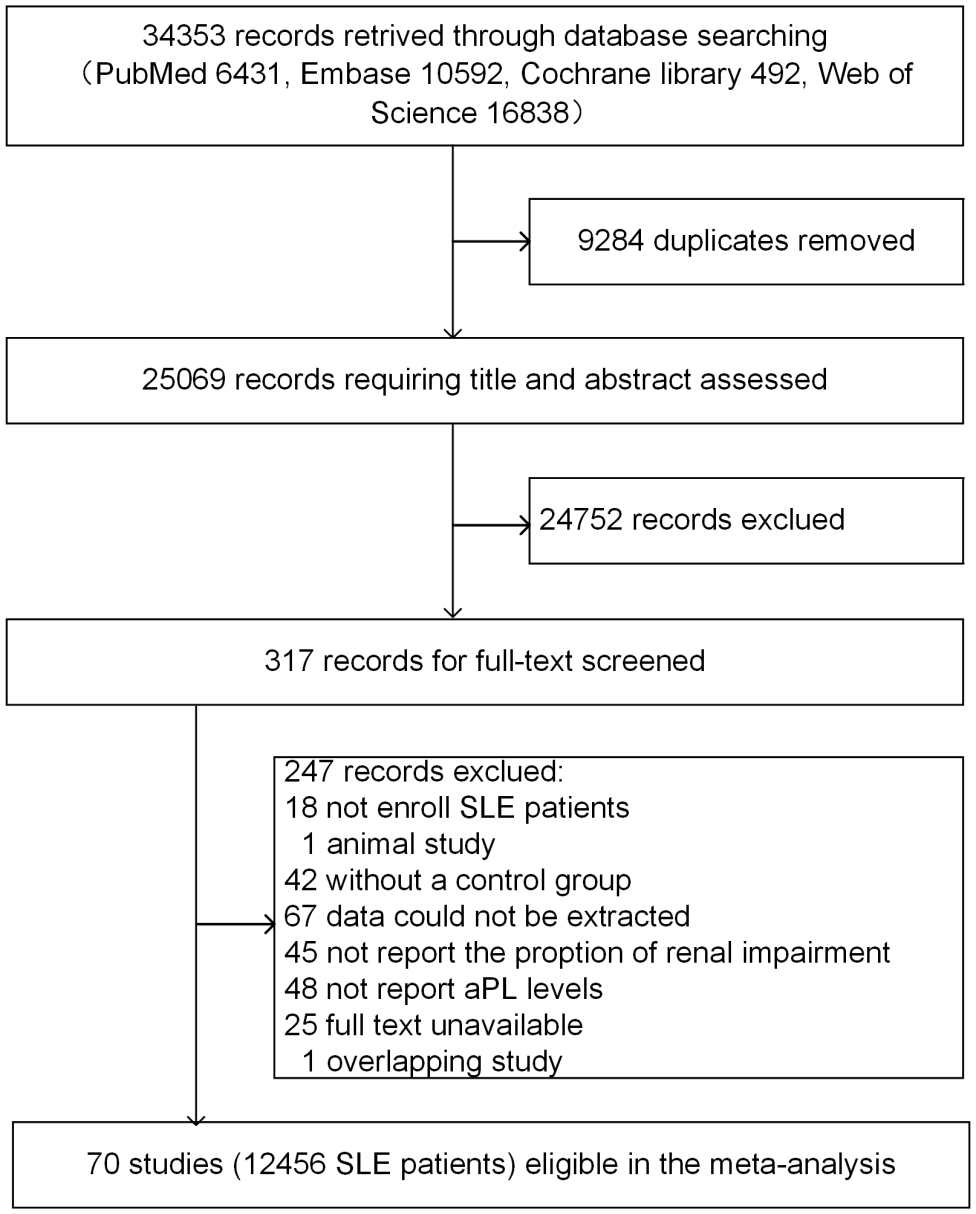
Flowchart of study identification and selection.

A total of 34,353 documents were retrieved up to September 12, 2025. Among them, 6,431 were from PubMed, 10,592 from Embase, 492 from Cochrane Library, and 16838 from Web of Science. After excluding 9,284 duplicates, the titles and abstracts of 25069 records were screened. A further 24,752 documents were excluded in terms of the exclusion criteria, leaving 317 studies to be read in full. Seventy studies comprising 12,456 samples were ultimately included after the exclusion of 18 studies that did not enroll SLE patients, 1 animal study, 42 studies without a control group, 67 studies from which data could not be extracted, 45 studies that did not report the number of renal impairment cases, 48 studies that did not report aPL levels, 25 studies for which full text was unavailable, and 1 overlapping study. **Figure 1** represented the direction of the literature search and study selection.

### Study characteristics

Of the 70 publications, 18 were case-control, 23 were cohort, and 29 studies were cross-sectional studies (13, 14, 21–88). Among them, four studies were multicenter, while sixty-six were conducted at a single center; Ten studies analyzed populations from the Americas, three from Africa, 22 from Asia, and 35 from Europe. The years of publication ranged from 1981 to 2025. Among the included 12,456 samples, the mean age ranged from 11 to 50.8 years. The baseline characteristics of the studies were listed in **Table 1**.

**Table 1:** Baseline characteristics of the original studies included in the meta-analysis.

Number	Source	Study type	Multicenter	Publication year	Inclusion period	Country	Location	Reported aPL	Sample size	Males (%): Females (%)	Age, y	Outcome
1	Helen I. Glueck et al.	Case-control	No	1985	1973.1-1982.1	USA	America	LA	77	12 (15.6%): 65 (84.4%)	NA	Renal vein thrombosis
2	K. E. Moss et al.	Cross-sectional	No	2001	1978.1.1-2000.7.1	Britain	Europe	APS	300	23 (7.7%): 277 (92.3%)	NA	Renal disease
3	P. Stratta et al.	Cross-sectional	No	1992	NA	Italy	Europe	LA	54	NA	NA	Thrombi
4	Taraneh Mehrani et al.	Cohort	No	2011	NA	USA	America	aβ2GPI IgM	796	63 (7.9%): 733 (92.1%)	45 (19.1-85.7)	Renal SLE
5	Ioannis Parodis et al.	Cross-sectional	No	2016	1995-2014	Sweden	Europe	aCL IgG IgM, APS, LA, aβ2GPI IgG IgM	498	70 (14.1%): 428 (85.9%)	NA	Renal SLE
6	Leyre Riancho-Zarrabeitia et al.	Cross-sectional	No	2020	2011.10-2012.9	Spain	Europe	aPL, APS	2398	230 (9.6%): 2168 (90.4%)	46.1 ± 14.2	Renal involvement
7	Savino Sciascia et al.	Cross-sectional	No	2020	NA	Italy	Europe	aCL, LA, aβ2GPI	124	28 (22.6%): 96 (77.4%)	44.5 ± 12.3	APLN
8	Małgorzata Wisłowska et al.	Cross-sectional	No	2014	2012-2013	Poland	Europe	aCL IgG IgM, APS, LA	100	8 (8%): 92 (92%)	38.9 ± 11.8	LN
9	Chindarat Natejumnong et al.	Cross-sectional	No	2006	2002.6-2003.12	Thailand	Asia	aCL IgG, LA, APS	77	3 (3.9%): 74 (96.1%)	NA	LN
10	S.Loizou et al. et al.	Cross-sectional	No	2000	NA	Britain	Europe	aCL overall/IgG/IgM, aβ2GPI overall/IgG/IgM	56	3 (5.4%): 53 (94.6%)	NA	LN
11	Juan Manuel Anaya et al.	Cross-sectional	Yes	2003	2002.1-2002.6	Colombia	America	aCL IgG IgM, LA	139	10 (7.2%): 129 (92.8%)	NA	LN
12	Maria G. Tektonidou et al.	Cross-sectional	No	2004	1987-2002	Greece	Europe	aPL, aCL, LA, APS	151	19 (12.6%): 132 (87.4%)	31.56 ± 13.18	APSN
13	Boonyarit Cheunsuchon et al.	Cross-sectional	No	2007	1999-2005	Thailand	Asia	APS	150	36 (24%): 114 (76%)	22.9 ± 11.4	APSN
14	Eric Daugas et al.	Cross-sectional	Yes	2002	1983-2000	France	Europe	aPL, APS, LA, aCL	96	NA	NA	APSN
15	Kotagal S. Kant et al.	Cohort	No	1981	1973.1-1979.4	USA	America	LA	105	NA	NA	Thrombi
16	Juan M. Miranda et al.	Cross-sectional	No	1994	NA	Mexico	America	aCL	36	NA	NA	Glomerular thrombosis
17	Ruitong Gao et al.	Cohort	No	2016	NA	China	Asia	aPL, APS	231	38 (16.5%): 193 (83.5%)	NA	APLN
18	Jakob Gerhardsson et al.	Cross-sectional	No	2015	1995.1-2012.12	Sweden	Europe	aCL, LA, aβ2GPI	112	23 (20.5%): 89 (79.5%)	38 (18-84)	APLN
19	Elena Gonzalo et al.	Cohort	Yes	2012	1986-2010	Spain	Europe	aPL	58	4 (6.9%): 54 (93.1%)	39 ± 12	Microthrombosis
20	Natasha Jordan et al.	Cohort	No	2014	1999-2012.2	England	Europe	aPL, APS	215	NA	NA	TMA
21	JM Mejıá-Vilet et al.	Cohort	No	2017	2008.1-2015.6	Mexico	Europe	aCL, LA, aβ2GPI, APS	353	NA	NA	TMA
22	Gabriella Moroni et al.	Cohort	No	2004	1990.1-2002.6	Italy	Europe	aPL, LA, aCL	111	10 (9%): 101 (91%)	28.5 ± 10.4	Renal insufficiency
23	R Shah et al.	Cohort	No	2018	2004.1-2015.12	USA	America	aPL	155	NA	NA	Renal zonal cortical scarring
24	Lihua Wu et al.	Cohort	No	2013	2000.1-2008.6	China	Asia	aCL	206	NA	NA	TMA
25	Ana Barrera-Vargas et al.	Case-control	No	2016	2008.1-2014.1	Mexico	Europe	aPL, APS	44	4 (9.1%): 40 (90.9%)	18-41	TMA
26	Emanuel Farrugia et al.	Case-control	No	1992	1964-1989	USA	America	LA	65	41 (63.1%): 24 (36.9%)	NA	TMA
27	Wafaa Gaber et al.	Case-control	No	2012	NA	Egypt	Africa	APS	64	0: 64 (100%)	NA	TMA
28	Indiran P. Naiker et al.	Cohort	No	2000	1996.9-1998.9	South Africa	Africa	aCL IgG	40	3 (7.5%): 37 (92.5%)	NA	Intrarenal microvascular thrombosis
29	S. Bhandari et al.	Cross-sectional	No	1998	NA	Britain	Europe	aCL	50	NA	NA	Thrombi
30	Danielle Cohen et al.	Cross-sectional	No	2008	1983-2006	The Netherlands	Europe	aPL	38	3 (7.9%): 35 (92.1%)	31.9 ± 11.2	Microthrombi
31	Gian Luca Erre et al.	Cross-sectional	No	2014	1986-2009	Italy	Europe	aPL, APS, LA, aCL	48	7 (14.6%): 41 (85.4%)	36 (14-66)	APSN
32	Marıá Galindo et al.	Cohort	No	2009	1986-2007	Spain	Europe	aPL, APS	65	6 (9%): 59 (91%)	42 ± 14	Microthrombi
33	Juan M. Miranda et al.	Cross-sectional	No	2009	NA	Mexico	Europe	aCL	162	18 (11.1%): 144 (88.9%)	27.6 ± 8.1	APSN
34	M. Perdiguero et al.	Cross-sectional	No	1995	NA	Spain	Europe	aPL	23	0: 23 (100%)	28.5 ± 12.3	Intraglomerular microthrombi
35	Y. Shen et al.	Cohort	No	2010	NA	China	Asia	aCL, LA, aβ2GPI	155	25 (16.1%): 130 (83.9%)	33 ± 13	Glomerular microthrombosis
36	R Silvariño et al.	Cohort	No	2011	1991-2008	Spain	Europe	aPL, LA, aCL IgG IgM	73	NA	NA	APLN
37	Di Song et al.	Cross-sectional	No	2013	2002.5-2008.7	China	Asia	aCL, aβ2GPI	148	26 (17.6%): 122 (82.4%)	NA	TMA
38	G. Hernández-Molina et al.	Cross-sectional	No	2015	2008-2012	Mexico	Europe	aPL, LA, aCL IgG IgM, aβ2GPI IgG IgM. APS	89	NA	NA	Chronic thrombotic microangiopathy (cTMA)
39	Faisal Naseeb et al.	Cross-sectional	No	2015	2013.1-2013.12	Saudi Arabia	Asia	APS	77	10 (13%): 67 (87%)	NA	APSN
40	Jianna Zhang et al.	Cross-sectional	No	2021	2017.1-2020.1	China	Asia	aPL	83	NA	NA	Microthrombosis
41	Hui Zheng et al.	Cohort	No	2009	2007.9-2008.10	China	Asia	aCL, LA, aβ2GPI	124	19 (15.3%): 105 (84.7%)	33 ± 14	Glomerular microthrombosis
42	Yan Zhou et al.	Cross-sectional	No	2019	2015.7-2018.7	China	Asia	aPL, LA, aCL overall/IgA IgG/IgM, aβ2GPI overall/IgA IgG/IgM	101	10 (9.9%): 91 (90.1%)	NA	Glomerular microthrombosis
43	D. P. D'CRUZ et al.	Cross-sectional	No	1991	NA	England	Europe	aCL	107	4 (3.7%): 103 (96.3%)	NA	LN
44	Geoffrey Frampton et al.	Case-control	No	1991	NA	England	Europe	aCL IgG	37	NA	NA	Intragiomerular thrombi
45	M Abu-Shakra et al.	Cohort	No	1996	1991.6-1994.5	Canada	America	aCL	23	5 (21.7%): 18 (78.3%)	28.3 (15-50)	Glomerular hyaline thrombi
46	Eric Descombes et al.	Cohort	No	1997	1980.1-1994.12	France	Europe	LA	123	NA	NA	Nonspecific sclerotic vascular lesions
47	M H Houman et al.	Case-control	No	2004	1987-2001	Tunisia	Africa	aCL, aβ2GPI	100	8 (8%): 92 (92%)	32	LN
48	Gözde Sevgi Kart Bayram et al.	Cross-sectional	No	2022	2019.10-2020.3	Turkey	Asia	APS	59	16 (27.1%): 43 (72.9%)	18-70	LN
49	Gudrun E Norby et al.	Cross-sectional	No	2010	1972-2005	Norway	Europe	aCL IgG IgM, LA	38	NA	NA	Recrudescent LN
50	E. Descloux et al.	Cohort	No	2008	1996-2006	France	Europe	aPL	55	NA	NA	Renal disorder
51	P Alba et al.	Case-control	No	2003	NA	Britain	Europe	aCL IgG IgM, LA	314	NA	NA	LN
52	D-C Varela et al.	Cross-sectional	No	2008	NA	Colombia	America	aCL IgG, APS, LA	365	NA	NA	LN
53	Yuki Tsuruta et al.	Case-control	No	2009	1995.1-2006.6	Japan	Asia	aPL	49	5 (10.2%): 44 (89.8%)	40.1 ± 14.3	CKD
54	Jing Luo et al.	Case-control	No	2004	NA	China	Asia	aCL IgG IgM, aβ2GPI IgG IgM	170	18 (10.6%): 152 (89.4%)	NA	LN
55	Xuhui Zhong et al.	Case-control	No	2011	1997.8-2009.12	China	Asia	aPL, aCL, LA, anti-β2GPI	44	21 (47.7%): 23 (52.3%)	NA	Glomerular microthrombus
56	Savino Sciascia et al.	Cohort	No	2021	NA	Italy	Europe	aPS/PT	52	10 (19.2%): 42 (80.8%)	NA	LN
57	Miaochen Yu et al.	Case-control	No	2022	2016.1-2017.4	China	Asia	aCL IgG IgM, aβ2GPI IgG IgM	116	19 (16.4%): 97 (83.6%)	NA	LN
58	Pei Zhang et al.	Case-control	No	2023	2017.1-2020.1	China	Asia	aCL, LA, aβ2GPI	191	29 (15.2%): 162 (84.8%)	NA	Glomerular microthrombosis
59	Bianka Perge et al.	Cohort	No	2024	1990-2020	Hungary	Europe	aCL, LA, aβ2GPI	384	45 (11.7%): 339 (88.3%)	50.8 ± 13..4	LN
60	Sulaiman M. Al-Mayouf et al.	Cohort	No	2023	1997.6-2022.7	Saudi Arabia	Asia	aPL	27	14 (51.9%): 13 (48.1%)	11 (8-16)	Renal involvement
61	Sulaiman M. Al-Mayouf et al.	Case-control	No	2015	2002.1-2014.6	Saudi Arabia	Asia	aPL	59	10 (16.9%): 49 (83.1%)	19.8 ± 4.4	Microthrombosis
62	Seoung Wan Nam et al.	Cohort	Yes	2018	2014.9-2015.12	Korea	Asia	aPL	469	33 (7%): 436 (93%)	NA	Renal disorder
63	Wanlin Cui et al.	Case-control	No	2015	2005.4-2013.10	China	Asia	aCL IgG IgM IgA	116	20 (17.2%): 96 (82.8%)	14.36 ± 2.05	LN
64	J. Font et al.	Cohort	No	2000	1988-1998	Spain	Europe	aPL, APS	100	10 (10%): 90 (90%)	NA	LN
65	WX Hu et al.	Case-control	No	2010	1987.6-2006.5	China	Asia	aCL	24	NA	NA	End-stage renal failure
66	Joanna Kosałka-Wegiel et al.	Case-control	No	2024	2012.1-2022.6	Poland	Europe	aCL overall/IgG/IgM, aβ2GPI overall/IgG/IgM, LA	921	102 (11.1%): 819 (88.9%)	NA	LN
67	Eman M. Farid et al.	Case-control	No	2013	1996-2012	Kingdom of Bahrain	Asia	aPL	88	6 (6.8%): 82 (93.2%)	NA	LN
68	M Dea´k et al.	Cohort	No	2014	NA	Hungary	Europe	APS	224	18 (8%): 204 (91%)	49 (20-92)	LN
69	A. Šipek-Dolničan et al.	Cross-sectional	No	2002	1988-1996	Slovenia	Europe	aCL	14	5 (35.7%): 9 (64.3%)	40.1 ± 14.7	Thrombi
70	Claire Barber et al.	Case-control	No	2012	1970-2007	Canada	America	APS	140	NA	NA	TMA

Underlined: aPL test included in the main analysis.

TMA,thrombotic microangiopathy; APSN, anti-phospholipid syndrome-associated nephritis;APLN, aPL-associated nephritis; SLE, systemic lupus erythematosus.

### Quality evaluation of included studies

The 70 studies had NOS scores ranging between 5 and 8, suggesting that their quality was appropriate for inclusion. The NOS scores for studies were shown in **Supplementary Tables S3**–**S5**.

### Risk of renal injury associated with aPL

**Figure 2:**
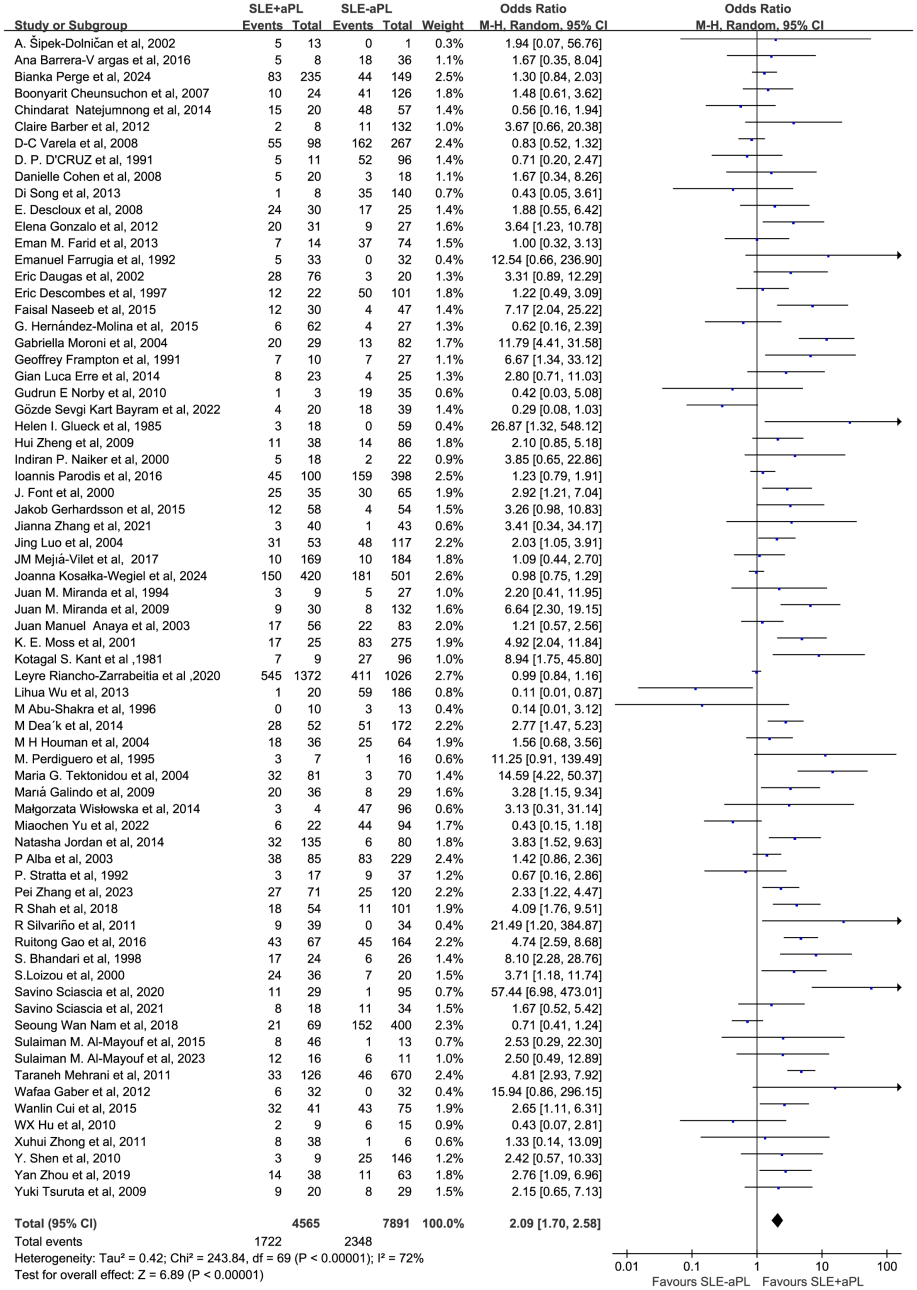
Forest plot for the association between aPL positivity and renal injury risk in SLE patients.

Renal injury developed in 1,722/4,565 (37.7%) of aPL-positive patients and 2,348/7,891 (29.8%) of aPL-negative patients in 70 studies involving 12,456 patients. The renal injury OR among aPL patients was 2.09 (1.70–2.58) as opposed to the aPL-negative patients (**Figure 2**). Among the 70 studies, 61 included SLE patients who underwent renal biopsy. The OR of aPL-positive group over aPL-negative group in this subgroup was 2.34 (1.85-2.97) (**Supplementary Figure S1**).

### Bias analysis

**Figure 3:**
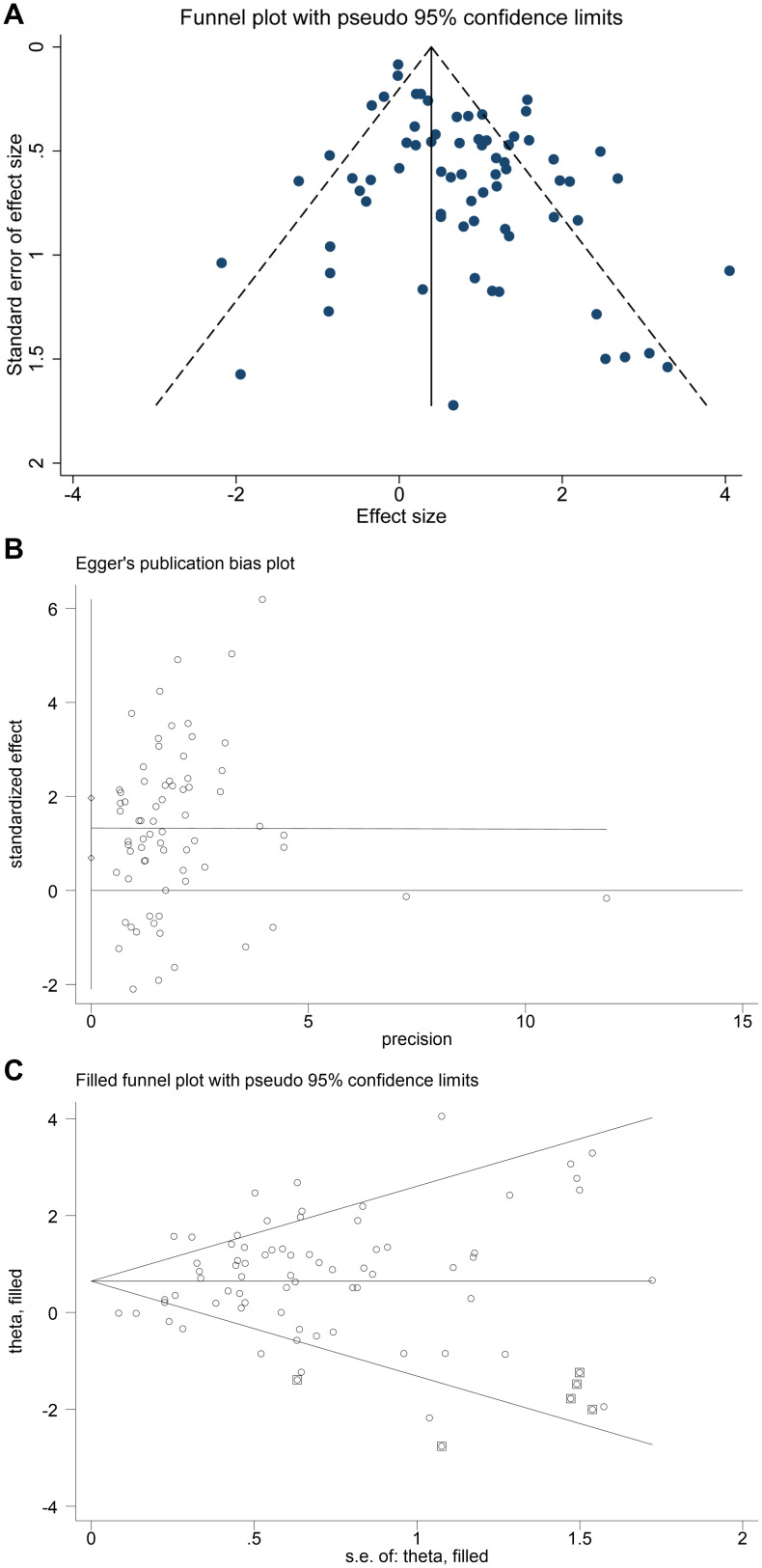
Publication bias assessment of the included studies. **(A)** Funnel plot. **(B)** Egger’s test plot. **(C)** Trim-and-fill analysis.

The asymmetry of the funnel plot suggests potential publication bias (**Figure 3A**), and the Egger’s test indicates that the meta-analysis may have publication bias (P < 0.001, **Figure 3B**). The trim−and−fill method was applied to adjust for potential publication bias. The results showed that, using a random-effects model for heterogeneity assessment, the pooled effect size (logOR) was 0.738 (0.528–0.948, P < 0.001). After five iterations, with six potentially missing studies imputed, the pooled effect size (logOR) was 0.646 (0.435–0.858, P < 0.001). The results remained statistically significant before and after the inclusion of the missing studies, indicating the robustness of the meta-analytic findings in the presence of publication bias. (**Figure 3C**).

### Sensitivity analysis

The sensitivity analysis, which entailed sequentially omitting one study at a time, indicated that our results were stable even in the presence of heterogeneity. As shown in **Supplementary Figure S2**, the pooled OR remained greater than 1 with a 95% CI excluding 1 in all iterations, indicating a robust association between aPL presence and increased risk of renal injury in SLE.

### Subgroup analysis

**Figure 4:**
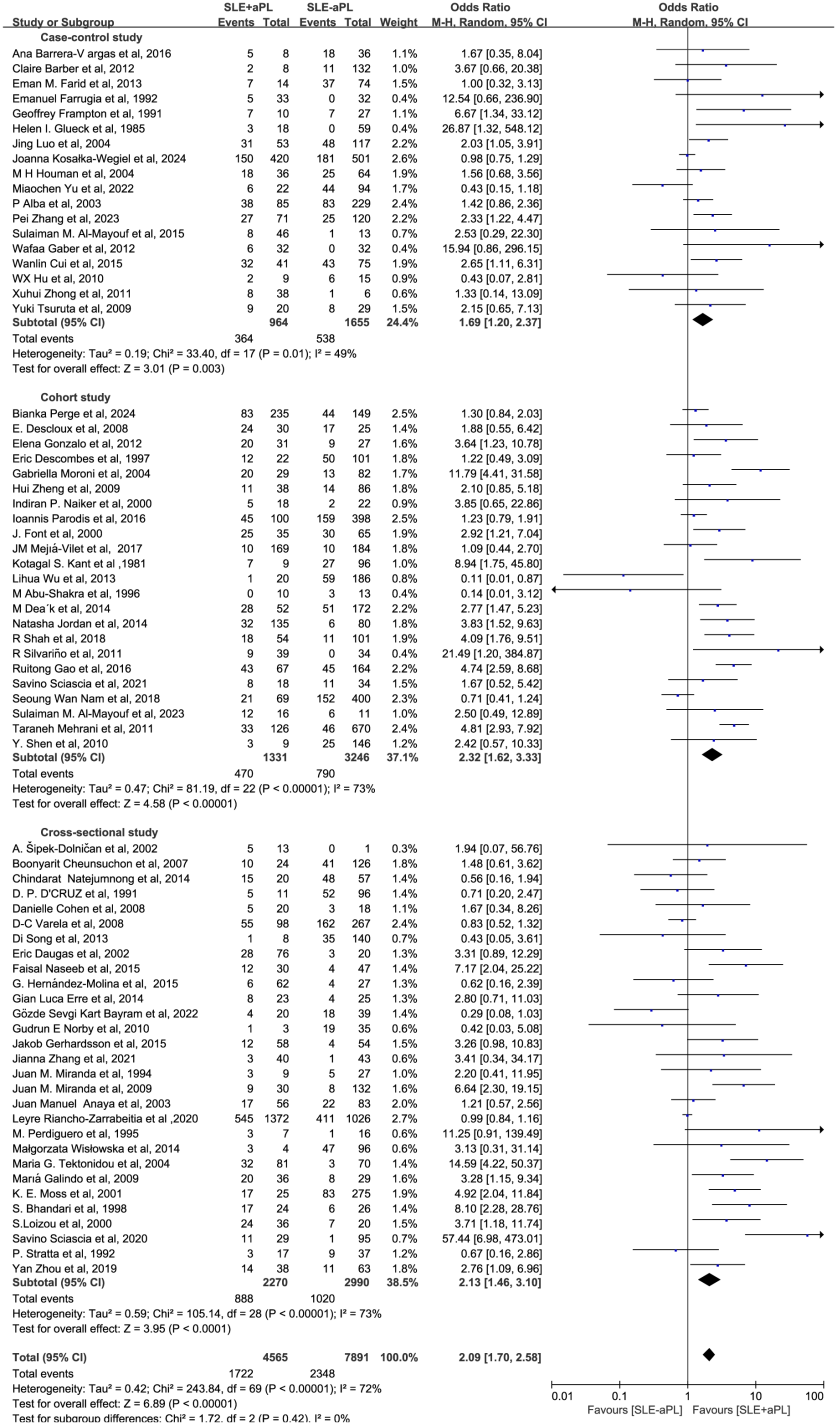
Subgroup analysis by study design: association of aPL positivity with renal injury risk.

Subgroup analyses were performed to explore potential sources of heterogeneity, stratified by study design, geographic location, publication period, aPL type, and pathological class of lupus nephritis. First, in terms of study design, studies of all types found that aPL positivity elevated the risk of renal injury in SLE patients (**Figure 4**). Across the included studies, renal injury incidence was as follows: in 18 case-control studies, 364/964 (37.8%) aPL-positive vs. 538/1,655 (32.5%) aPL-negative patients; in 23 cohort studies, 470/1331 (35.3%) aPL-positive vs. 790/3246 (24.3%) aPL-negative patients; and in 29 cross-sectional studies, 888/2,270 (39.1%) aPL-positive vs. 1,020/2,990 (34.1%) aPL-negative patients. The OR for case-control, cohort, and cross-sectional studies were 1.69 (1.20-2.37), 2.32 (1.62-3.33), and 2.13 (1.46-3.10), respectively. Further, we performed the meta-analysis of relative risk (RR) in the cohort study subgroup to give a more precise estimate of the actual effect size. The meta-analysis of 23 cohort studies indicated that the risk of renal impairment was considerably greater in the group of patients who had aPL than in the group without aPL, with an RR of 1.71 (1.37–2.13) (**Supplementary Figure S3**).

**Figure 5:**
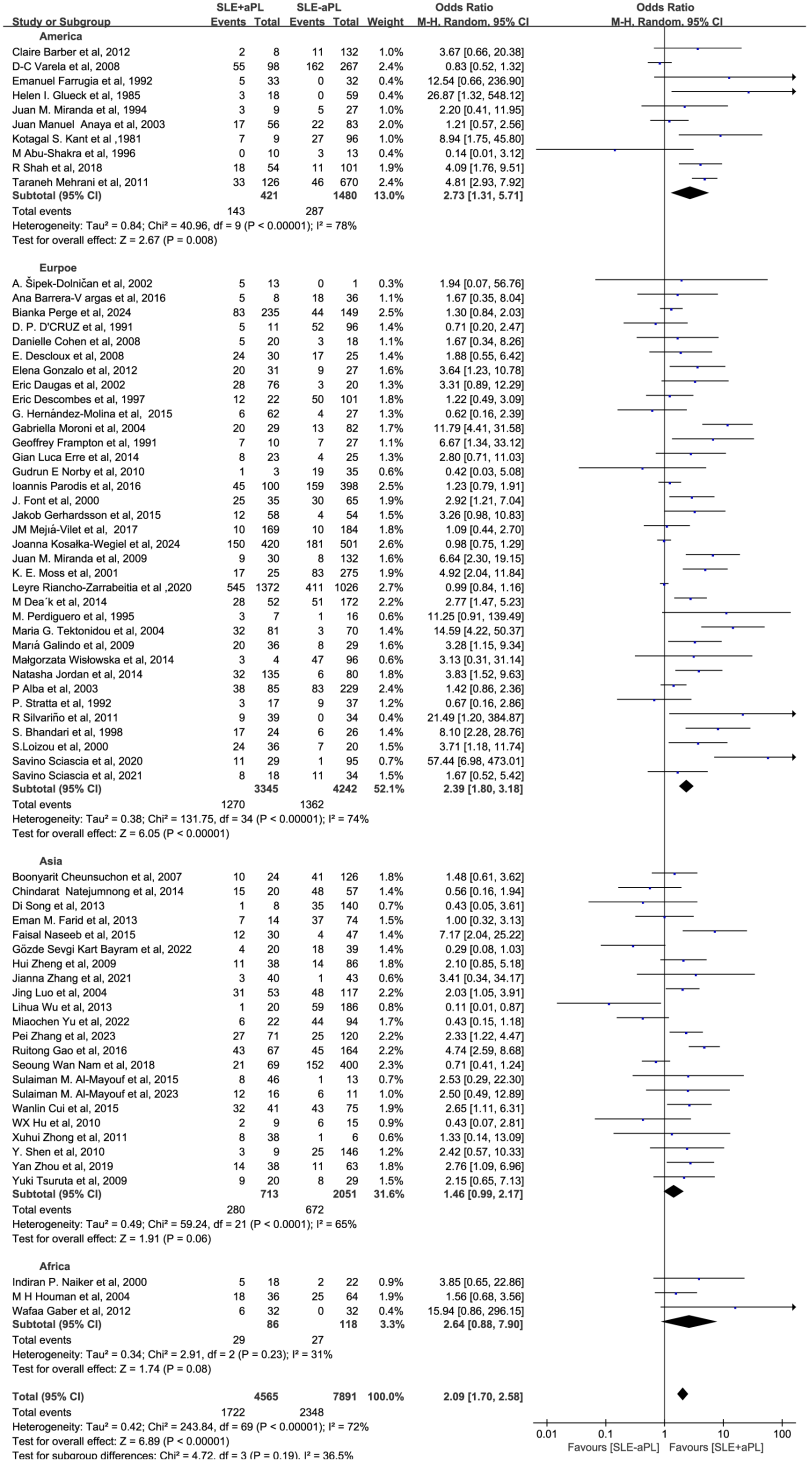
Subgroup analysis by geographical region: association of aPL positivity with renal injury risk.

Subgroup analysis by geographical region revealed non-significant associations between aPL and renal injury in Asia and Africa (OR of 1.46 [0.99–2.17] and 2.64 [0.88-7.90], respectively), whereas significant positive associations were found in Europe and America (OR of 2.39 [1.80–3.18] and 2.73 [1.31–5.71], respectively; **Figure 5**).

**Figure 6:**
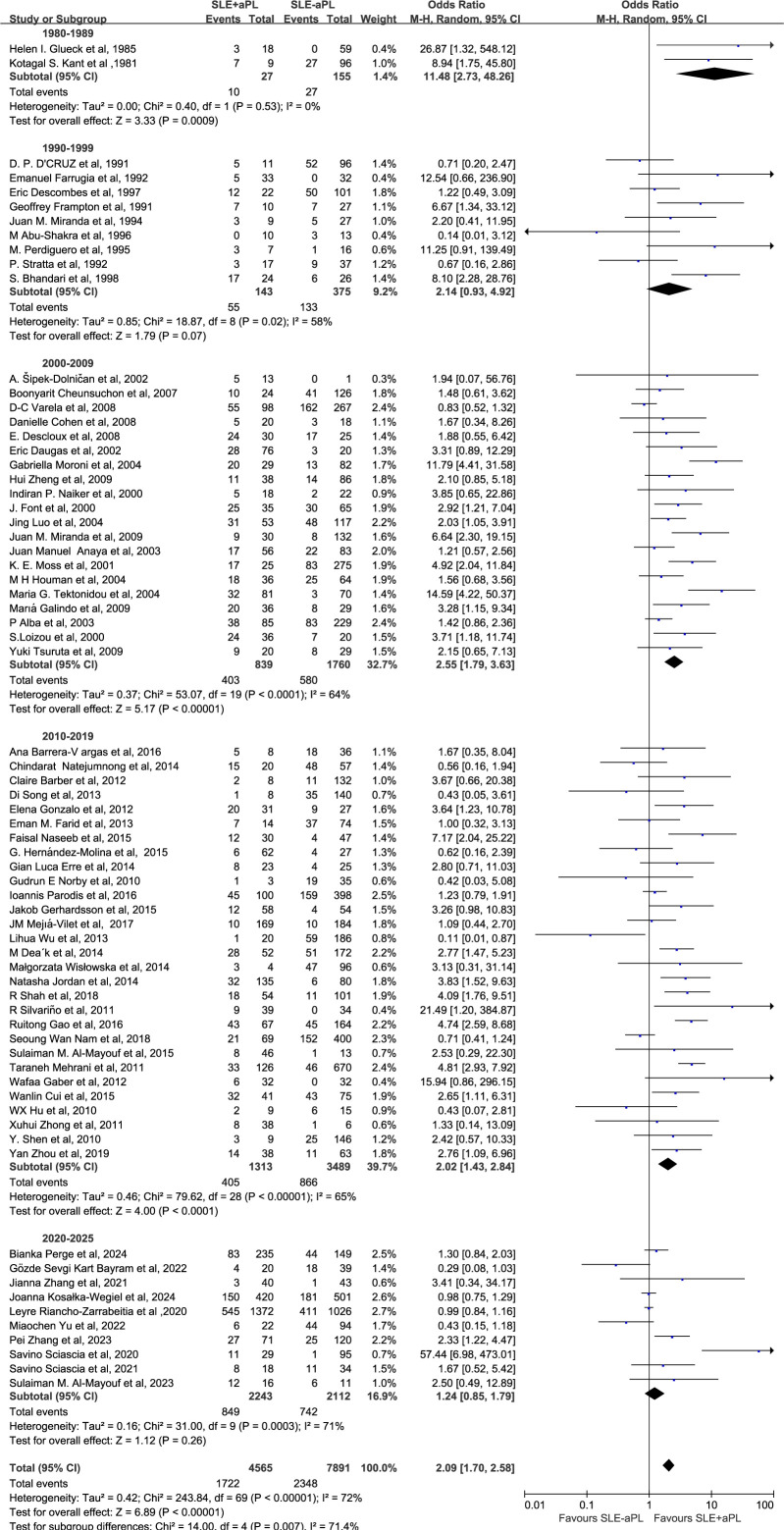
Subgroup analysis by publication period: association of aPL positivity with renal injury risk.

Subgroup analysis by publication periods demonstrated OR of 11.48 (2.73–48.26) for 1980-1989, 2.14 (0.93-4.92) for 1990-1999, 2.55 (1.79–3.63) for 2000-2009, 2.02 (1.43–2.84) for 2010-2019, and 1.24 (0.85–1.79) for 2020-2025 (**Figure 6**).

**Figure 7:**
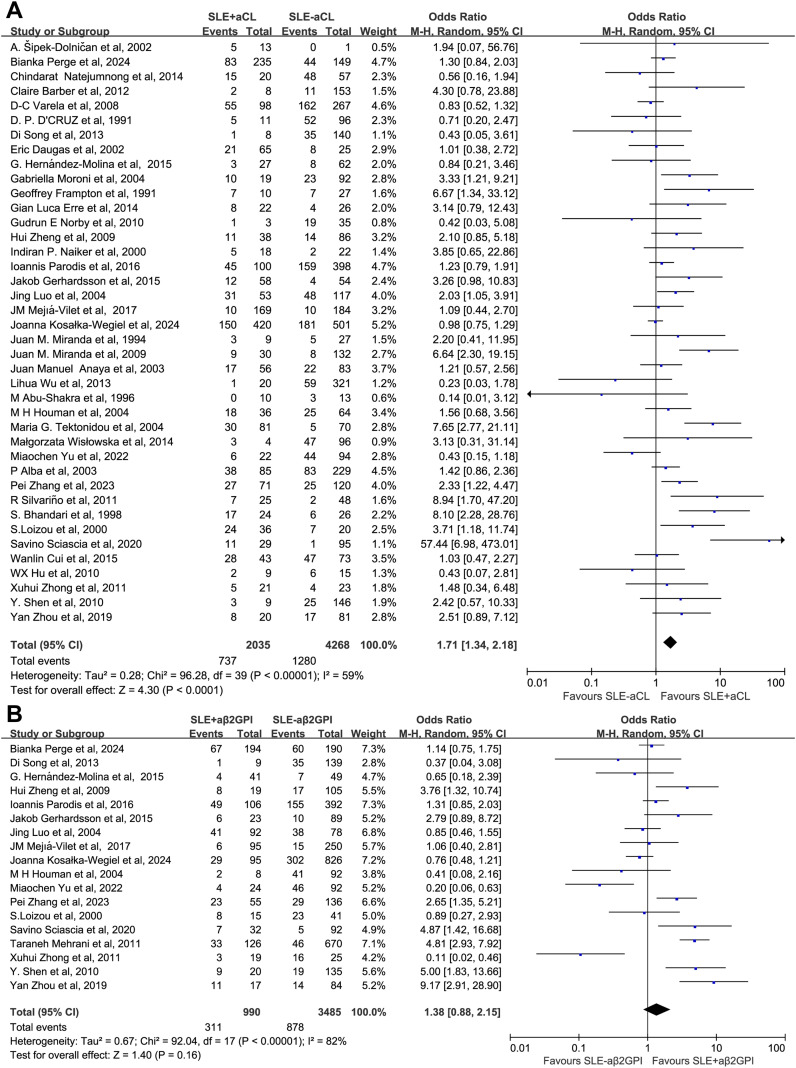
Subgroup analysis by aPL category. **(A)** Overall aCL. **(B)** Overall aβ2GPI.

**Figure 8:**
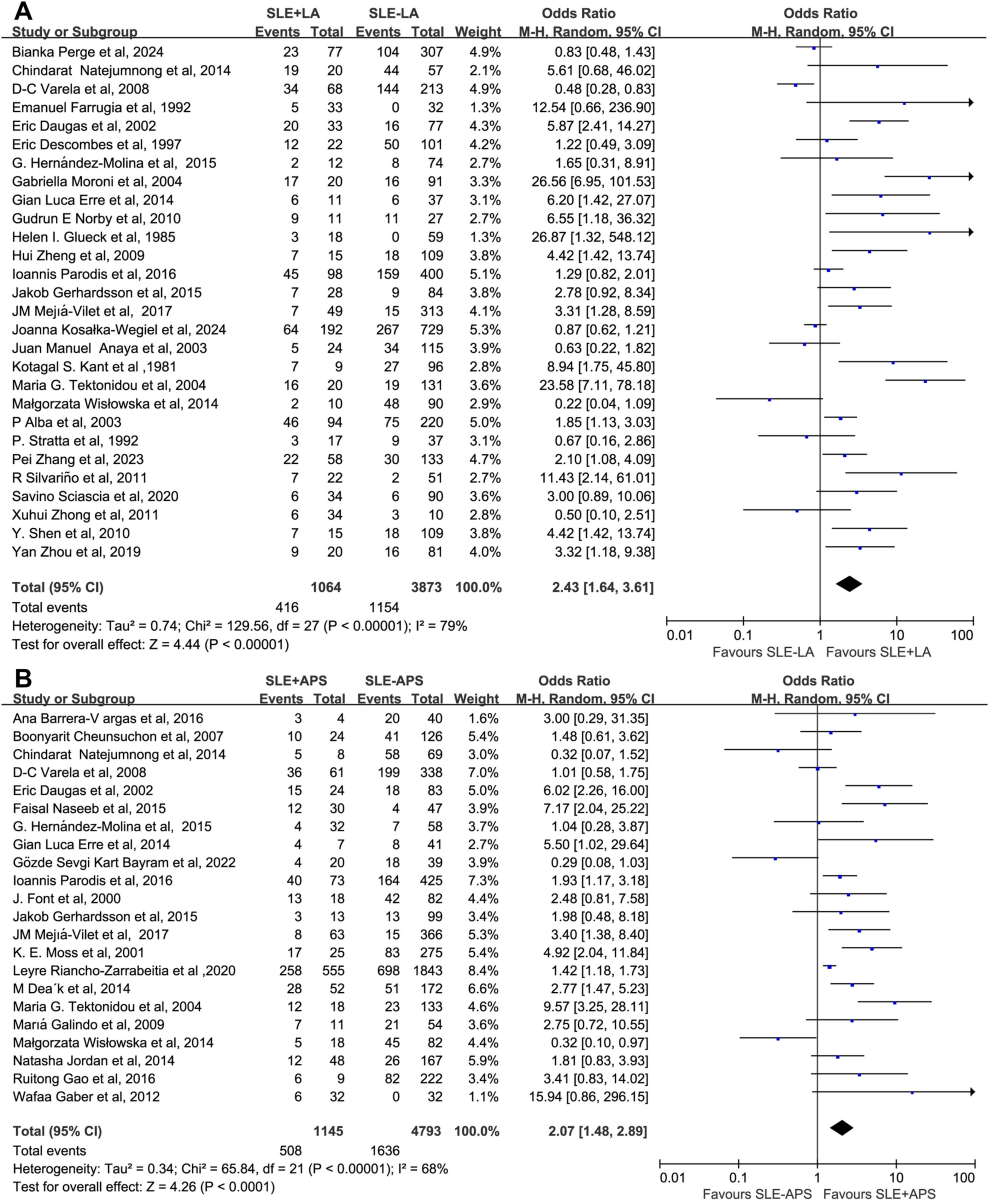
Subgroup analysis by aPL type. **(A)** LA. **(B)** APS.

The association between different aPL types and renal injury was further analyzed. The presence of aCL, LA, and APS was associated with a significantly increased risk of renal injury compared with the control group, with OR of 1.71 (1.34–2.18), 2.43 (1.64–3.61), and 2.07 (1.48–2.89), respectively. In contrast, aβ2GPI showed no significant difference, with an OR of 1.38 (0.88–2.15) (**Figures 7**, **8**). Further analysis of the associations between the three aCL subtypes (IgG, IgM, and IgA) and renal injury revealed OR of 1.51 (1.11–2.06), 0.81 (0.66-1.00), and 1.87 (0.82–4.27), respectively, suggesting that the aCL IgG subtype primarily promotes renal injury (**Supplementary Figure S4**). Neither aβ2GPI nor its isotypes conferred a significant increase in renal injury risk. The OR for the IgG isotype of aβ2GPI was 1.24 (0.71–2.18), while that for the IgM isotype was 1.18 (0.52–2.69), and the OR for the IgA isotype was 5.05 (0.79–32.13) (**Supplementary Figure S5**). Regarding aPS/PT, data from a single study also showed no significant association (OR 1.67, 0.52–5.42) (**Supplementary Figure S6**). Finally, subgroup analyses revealed no significant association between aPL status and the histopathological classification (except for class II) of lupus nephritis. The OR for aPL positivity across class I-VI lupus nephritis were 0.37 (0.05–2.93), 1.53 (1.02–2.28), 1.21 (0.88–1.67), 1.27 (0.81–1.98), 0.98 (0.70–1.36), and 0.80 (0.23–2.87), respectively (**Supplementary Figures S7**, **S8**).

### Cumulative meta-analysis

**Figure 9:**
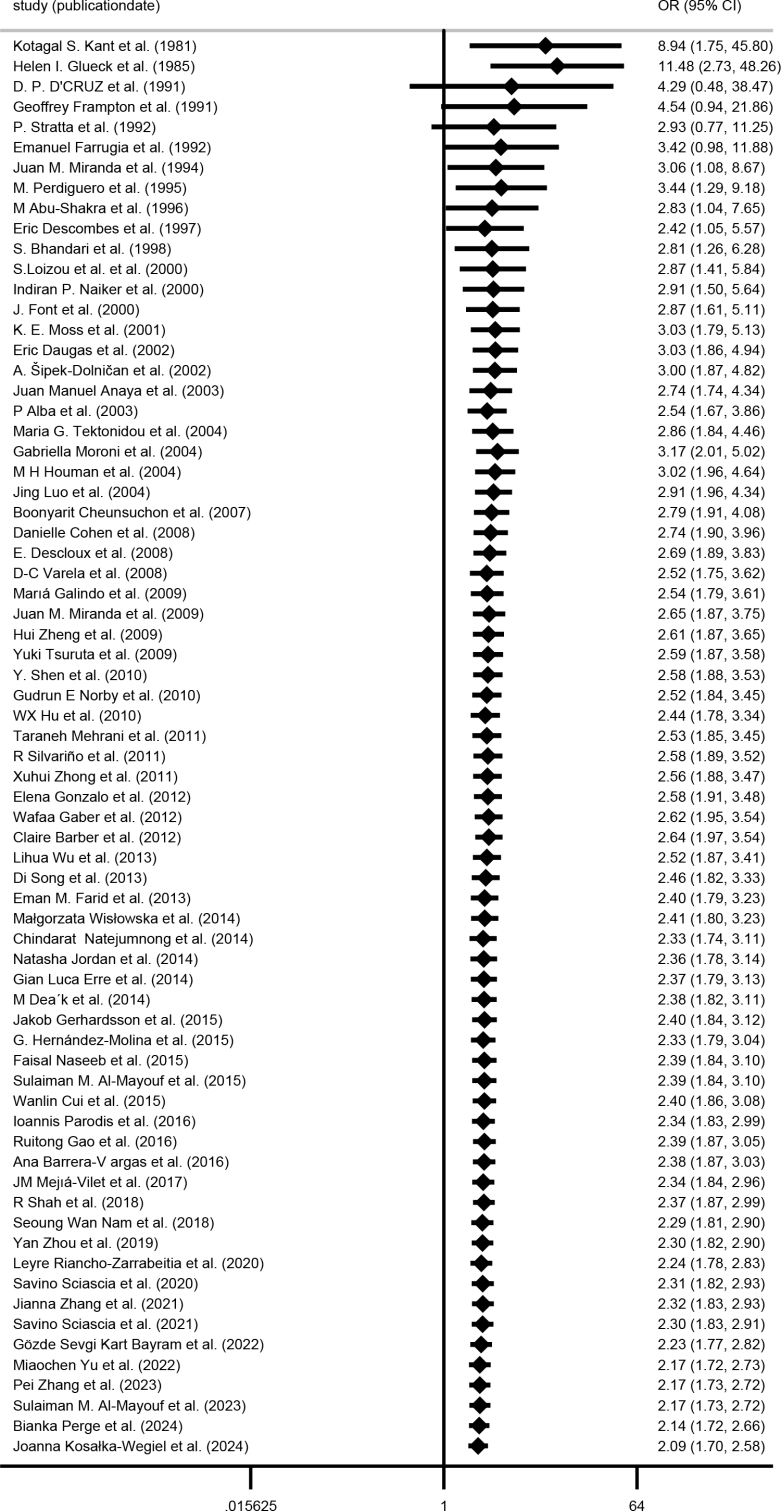
Cumulative meta-analysis sorted by publication date.

**Figure 10:**
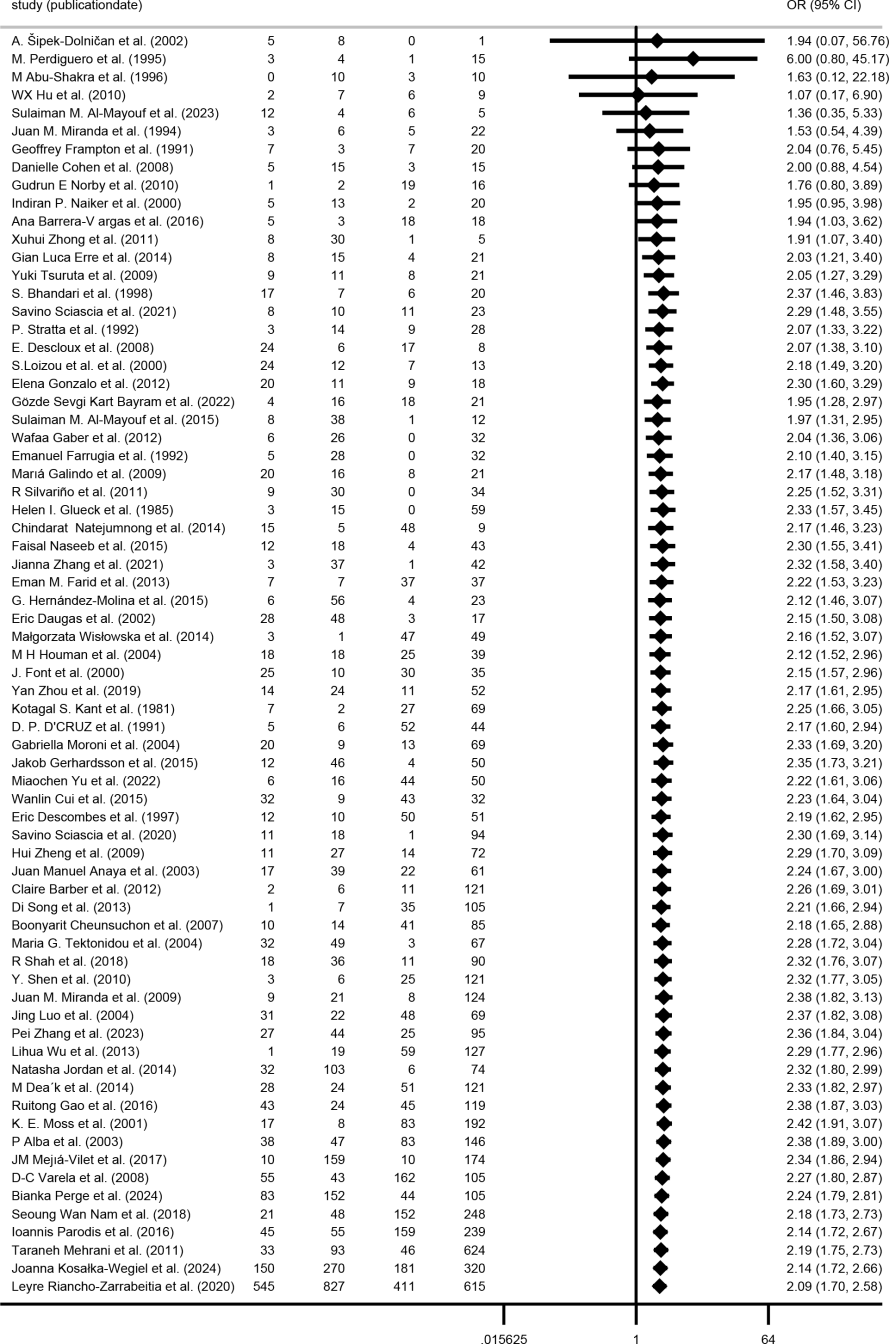
Cumulative meta-analysis sorted by sample size.

**Figure 11:**
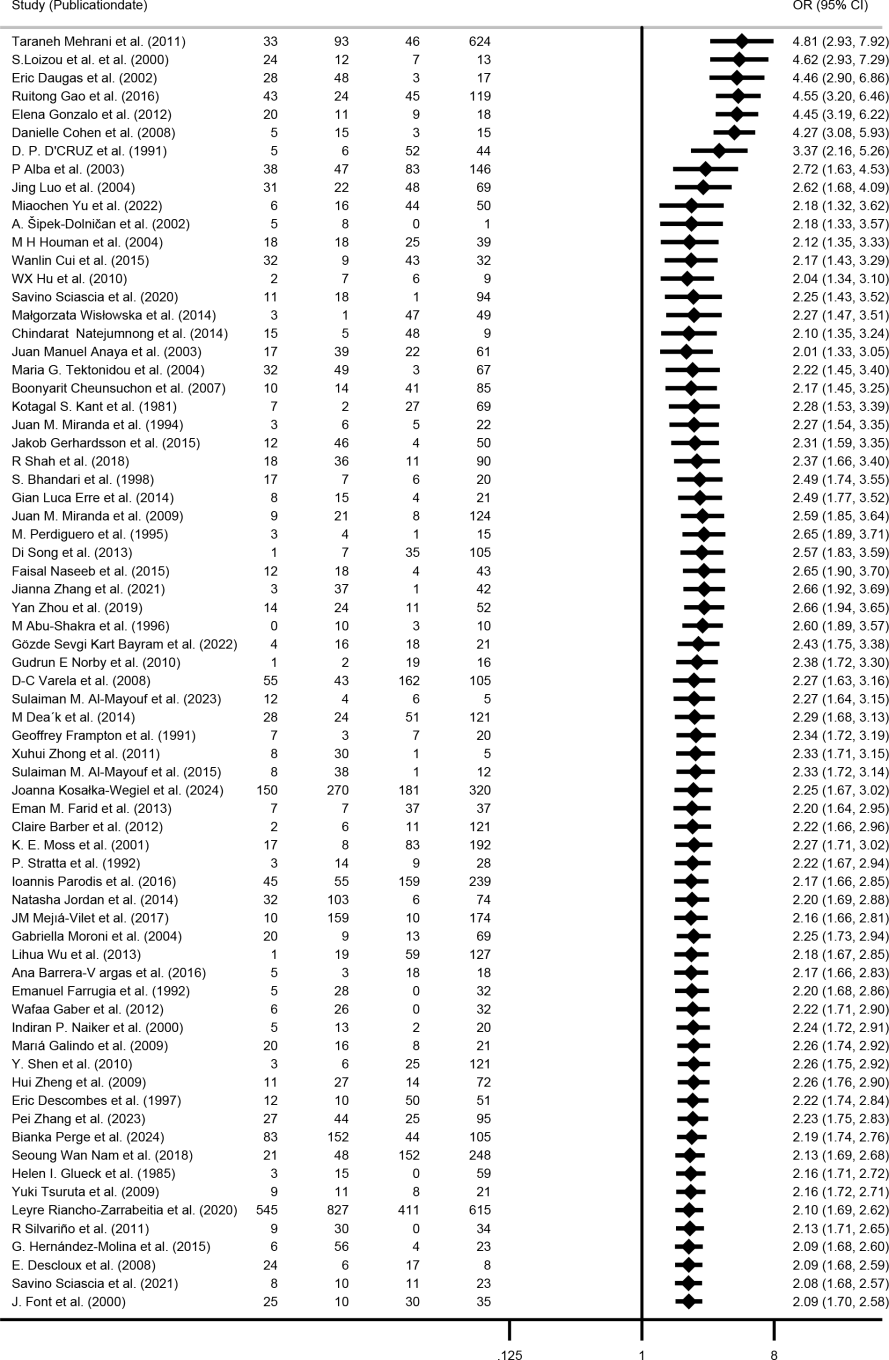
Cumulative meta-analysis sorted by study quality (NOS score).

Three separate cumulative analyses were performed by sequentially adding studies based on publication date, sample size, and NOS. These analyses consistently revealed an elevated kidney injury risk in the aPL-positive group. As evidence accumulated, the 95% CI progressively narrowed, and the point estimates converged, indicating enhanced precision and result stability (**Figures 9**–**11**).

## Discussion

To elucidate the association between aPL profiles and renal injury in SLE, this meta-analysis evaluated 70 studies involving 12,456 patients. The results collectively provide compelling evidence that aPL seropositivity confers a significantly increased risk of renal injury. These findings carry important implications for clinical practice, suggesting that aPL status may serve as a key indicator for renal injury risk stratification and monitoring in SLE.

Immune complex deposition, complement activation, and recruitment of pro-inflammatory cells are central mechanisms in the pathogenesis of kidney injury in SLE (89). The aPL-associated renal disease represents a heterogeneous group of conditions with various clinical manifestations (90). Vascular involvement in aPL−related renal disease may manifest as thrombosis, stenosis, or infarction. Clinically, these lesions can present with proteinuria, hematuria, hypertension, acute kidney injury, or chronic kidney disease. The mechanisms by which aPL induce renal injury in patients with SLE remain unclear. Chul-Soo Cho et al. found that aCL can stimulate endothelial cells to secrete monocyte chemoattractant protein-1, thereby recruiting monocytes (91). Additionally, aCL cross-reacts with oxidized low-density lipoprotein, promoting increased cholesterol uptake by macrophages and their transformation into foam cells, which contributes to the development of atherosclerosis (92). Shengshi Huang et al. found that aPL bind to extroverted lipids on the surface of platelets, promote platelet activation, participate in coagulation cascades, and cause thrombosis (93). As shown in one of our previous studies, aPS IgG antibodies form PS-IgG immune complexes with PS antigen which stimulate the oxidative stress in macrophages in a LOX-dependent manner, impairs phagocytic activity, and finally results in the lupus nephritis development (94). These findings collectively indicate that aPL primarily promotes nephritis pathogenesis through inducing renal vascular thrombosis and immune dysregulation.

Our findings suggest that aPL may serve as a predictive biomarker for renal injury in lupus. Renal biopsy is generally considered the standard for diagnosing and classifying types of renal injury. However, renal biopsy is an invasive procedure; it is associated with complications such as hematuria, hematoma, back pain, bleeding and fever. A number of laboratory and clinical characteristics were observed to aid in early detection of patients at high risk of developing lupus nephritis. The biomarkers used in the diagnosis process and in the prognosis prediction are: complement, autoantibodies (anti-double−stranded DNA antibodies, anti-chromatin antibodies), cytokines (monocyte chemoattractant protein-1, type I and type II interferons), genetic defects (DNA/RNA clearance, complement pathway, anti-nucleic acid sensing in interferon pathway) (95). SLE patients show immune dysfunction, loss of immune tolerance, exposure of phospholipids on the outer leaflet of the plasma membrane, and hyperactivity of B-cells, which leads to high levels of production of autoantibodies (96). The immune complex development of the antigen-antibody complexes in the kidney results in the release of complement, activation of pro-inflammatory cells, and release of pro-inflammatory cytokines, which cause renal injury (97). Multiple studies have reported that aPL positivity is a major risk factor for kidney injury in patients with SLE. Therefore, coagulation parameters and renal function should be closely monitored in aPL−positive SLE patients.

Substantial heterogeneity was observed across studies (I² > 50%), which justified the use of random−effects models. To explore the cause of this heterogeneity, subgroup analyses showed that the strength of the association between aPL and renal injury is significantly different based on region, period of publication, and type of aPL, suggesting these factors as likely contributors to the heterogeneity. Furthermore, both funnel plot and Egger’s test indicated publication bias. Notwithstanding these issues, sensitivity analysis alongside the trim-and-fill method consistently demonstrated a significant risk increase, affirming the robustness of the primary finding that aPL positivity elevates renal injury risk in SLE patients. In addition, we performed cumulative analyses by sequentially adding each new study according to publication year, sample size, and NOS score, thereby dynamically illustrating the changing trend of the research findings. The results consistently showed a gradual narrowing of the 95% CI of the OR values, suggesting the robustness of the main findings of this study.

An earlier meta-analysis conducted by Vinicius Domingues et al. with 35 studies and 3,035 SLE patients up to 2021 showed that aPL-positive patients had a 3.03-fold increased risk of renal microthrombotic lesions (18). This study conducted a comprehensive literature search up to the association between aPL and renal injury up to September 2025 to compute 70 studies that included 12,456 participants. In aPL positive and aPL negative SLE, the pooled OR of renal microvascular lesions was that 2.09 (1.70–2.58). Some studies have revealed that non-criteria aPL, such as aPS, aPT, and aPS/PT antibodies, are also involved in SLE-associated renal injury (96). The aPL that are tested clinically mainly belong to the IgG and IgM isotypes, but IgA antibodies have also been implicated in SLE pathogenesis (97). Therefore, we also focused on the roles of different antibody isotypes (IgG/IgM/IgA) and non-criteria aPL subtypes in various types of aPL-related kidney involvement (such as lupus nephritis and its different classes, chronic kidney disease, renal microvascular thrombosis, and end-stage renal disease), thereby supplementing and updating current research on aPL-related renal injury. Although the meta-analysis included the novel aPL subtypes described above in the search strategy, only two studies reported on aCL IgA, one study investigated aβ2GPI IgA, and one study examined aPS/PT antibodies. The limited number of studies may explain why no significant association was found between these novel antibodies and renal injury. More clinical and basic studies are required to determine the roles of novel aPL and their different isoforms in kidney injury of SLE patients.

The research has several shortcomings. To begin with, there is a difference in the modes of detection of aPL between the 70 studies. aCL and aβ2GPI levels are typically enzyme-linked immunosorbent assays, whereas LA are typically determined by the activated partial thromboplastin time, diluted Russell viper venom time, or silica clotting time. It is also difficult to establish a diagnostic standard for positive aPL levels due to differences in kits, instruments, and positive cutoff values. These differences in assay methods present challenges for the combination of data from different studies. As expected, significant heterogeneity were observed across the included studies. Second, not all the studies was used because the full text was not available. Thus, this meta-analysis did not involve all possible studies. Moreover, since various study design (cohort, cross-sectional, and case-control studies) were involved in this meta-analysis, we could only use OR as the pooled effect measure. As the meta-analysis assigns different weights to individual studies according to their variance, the pooled OR of 2.09 may deviate to some extent from the true effect. To better reflect the actual magnitude of the association, we thus did another meta-analysis in RR limited to cohort studies. The outcome of 23 cohort studies revealed that the risk of renal impairment was notably greater in the aPL-positive cohort compared to the aPL-negative counterpart with the total RR of 1.71 (1.37–2.13). In addition, the 70 studies involved in this meta-analysis cover a considerable time frame (1981–2024), which inevitably made the diagnosis criteria of SLE not consistent, as it embraced the American College of Rheumatology (ACR; 1971, 1982, 1997, 2009, and 2012), the Systemic Lupus International Collaborating Clinics (SLICC 2012) classification criteria, and the European League Against Rheumatism/American College of Rheumatology (2019 EULAR/ACR) criteria, which may affect the reliability and comparability of the results.

It is worth noting that retrospective studies are inevitably subject to information bias, selection bias, and confounding bias due to the limitations of the type of studies to be included in this study. As such, the meta-analysis can only conclude that patients with SLE who are aPL−positive have a higher risk of kidney injury. In the absence of greater evidence of large perspective randomized controlled trials, whether aPL−positive patients remain at a higher risk of kidney injury despite such treatment and subsequent follow-ups remain open. Moreover, in the absence of cellular and animal experimental data, we were unable to further explore the mechanisms by which aPL mediate renal injury in SLE. In future more clinical trials and experimental experiments would be required to further affirm the role and mechanism of aPL in kidney injury.

## Conclusions

SLE patients with positive aPL (LA and aCL) or secondary APS are at significantly higher risk of renal injury than those who are aPL-negative. Risk stratification of different aPL types may facilitate clinical management in patients with SLE.

## Data Availability

The original contributions presented in the study are included in the article/**Supplementary Material**. Further inquiries can be directed to the corresponding author/s.
