# Supplementary material for: Anti-phospholipid antibodies as a risk factor for renal injury in patients with systemic lupus erythematosus: a comprehensive analysis
Source: Front Immunol. 2026 Feb 3;17:1734274. doi: 10.3389/fimmu.2026.1734274 (PMC12909180; doi:10.3389/fimmu.2026.1734274)
Supplement: Supplementary file 1 [file DataSheet1.docx]

**Supplementary materials**

**Anti-phospholipid antibodies as a risk factor for renal injury in patients with systemic lupus erythematosus: a comprehensive analysis**

Inventory of supplementary data

1. Table S1………….………………………… Page 2
2. Table S2………….………………………… Page 6
3. Table S3………….………………………… Page 9
4. Table S4………….………………………… Page 10
5. Table S5………….………………………… Page 12
6. Figure S1………….…………………………Page 14
7. Figure S2………….…………………………Page 15
8. Figure S3………….…………………………Page 16
9. Figure S4………….…………………………Page 17
10. Figure S5………….…………………………Page 18
11. Figure S6………….…………………………Page 19
12. Figure S7………….…………………………Page 20
13. Figure S8………….…………………………Page 21

## Table S1. PRISMA checklist.

| **Section and Topic** | **Item #** | **Checklist item** | **Location where item is reported** |
| --- | --- | --- | --- |
| **TITLE** | | |  |
| Title | 1 | Identify the report as a systematic review. | 1 |
| **ABSTRACT** | | |  |
| Abstract | 2 | See the PRISMA 2020 for Abstracts checklist. | 1-2 |
| **INTRODUCTION** | | |  |
| Rationale | 3 | Describe the rationale for the review in the context of existing knowledge. | 2 |
| Objectives | 4 | Provide an explicit statement of the objective(s) or question(s) the review addresses. |  |
| **METHODS** | | |  |
| Eligibility criteria | 5 | Specify the inclusion and exclusion criteria for the review and how studies were grouped for the syntheses. | 3 |
| Information sources | 6 | Specify all databases, registers, websites, organisations, reference lists and other sources searched or consulted to identify studies. Specify the date when each source was last searched or consulted. | 3 |
| Search strategy | 7 | Present the full search strategies for all databases, registers and websites, including any filters and limits used. | Page 6-8 of supplemental material |
| Selection process | 8 | Specify the methods used to decide whether a study met the inclusion criteria of the review, including how many reviewers screened each record and each report retrieved, whether they worked independently, and if applicable, details of automation tools used in the process. | 3 |
| Data collection process | 9 | Specify the methods used to collect data from reports, including how many reviewers collected data from each report, whether they worked independently, any processes for obtaining or confirming data from study investigators, and if applicable, details of automation tools used in the process. | 3 |
| Data items | 10a | List and define all outcomes for which data were sought. Specify whether all results that were compatible with each outcome domain in each study were sought (e.g. for all measures, time points, analyses), and if not, the methods used to decide which results to collect. | 3 |
|  | 10b | List and define all other variables for which data were sought (e.g. participant and intervention characteristics, funding sources). Describe any assumptions made about any missing or unclear information. | 3 |
| Study risk of bias assessment | 11 | Specify the methods used to assess risk of bias in the included studies, including details of the tool(s) used, how many reviewers assessed each study and whether they worked independently, and if applicable, details of automation tools used in the process. | 3 |
| Effect measures | 12 | Specify for each outcome the effect measure(s) (e.g. risk ratio, mean difference) used in the synthesis or presentation of results. | 4 |
| Synthesis methods | 13a | Describe the processes used to decide which studies were eligible for each synthesis (e.g. tabulating the study intervention characteristics and comparing against the planned groups for each synthesis (item #5)). | 3 |
|  | 13b | Describe any methods required to prepare the data for presentation or synthesis, such as handling of missing summary statistics, or data conversions. | 3 |
|  | 13c | Describe any methods used to tabulate or visually display results of individual studies and syntheses. | 4 |
|  | 13d | Describe any methods used to synthesize results and provide a rationale for the choice(s). If meta-analysis was performed, describe the model(s), method(s) to identify the presence and extent of statistical heterogeneity, and software package(s) used. | 3 |
|  | 13e | Describe any methods used to explore possible causes of heterogeneity among study results (e.g. subgroup analysis, meta-regression). | 3 |
|  | 13f | Describe any sensitivity analyses conducted to assess robustness of the synthesized results. | 3 |
| Reporting bias assessment | 14 | Describe any methods used to assess risk of bias due to missing results in a synthesis (arising from reporting biases). | 3 |
| Certainty assessment | 15 | Describe any methods used to assess certainty (or confidence) in the body of evidence for an outcome. | 3 |
| **RESULTS** | | |  |
| Study selection | 16a | Describe the results of the search and selection process, from the number of records identified in the search to the number of studies included in the review, ideally using a flow diagram. | 4 |
|  | 16b | Cite studies that might appear to meet the inclusion criteria, but which were excluded, and explain why they were excluded. | 4 |
| Study characteristics | 17 | Cite each included study and present its characteristics. | Page 4 and table 1 |
| Risk of bias in studies | 18 | Present assessments of risk of bias for each included study. | 4 |
| Results of individual studies | 19 | For all outcomes, present, for each study: (a) summary statistics for each group (where appropriate) and (b) an effect estimate and its precision (e.g. confidence/credible interval), ideally using structured tables or plots. | 4 |
| Results of syntheses | 20a | For each synthesis, briefly summarise the characteristics and risk of bias among contributing studies. | 4 |
|  | 20b | Present results of all statistical syntheses conducted. If meta-analysis was done, present for each the summary estimate and its precision (e.g. confidence/credible interval) and measures of statistical heterogeneity. If comparing groups, describe the direction of the effect. | 4 |
|  | 20c | Present results of all investigations of possible causes of heterogeneity among study results. | 5 |
|  | 20d | Present results of all sensitivity analyses conducted to assess the robustness of the synthesized results. | 4 |
| Reporting biases | 21 | Present assessments of risk of bias due to missing results (arising from reporting biases) for each synthesis assessed. | 4 |
| Certainty of evidence | 22 | Present assessments of certainty (or confidence) in the body of evidence for each outcome assessed. | 4 |
| **DISCUSSION** | | |  |
| Discussion | 23a | Provide a general interpretation of the results in the context of other evidence. | 11 |
|  | 23b | Discuss any limitations of the evidence included in the review. | 15 |
|  | 23c | Discuss any limitations of the review processes used. | 15 |
|  | 23d | Discuss implications of the results for practice, policy, and future research. | 16 |
| **OTHER INFORMATION** | | |  |
| Registration and protocol | 24a | Provide registration information for the review, including register name and registration number, or state that the review was not registered. | 2 |
|  | 24b | Indicate where the review protocol can be accessed, or state that a protocol was not prepared. | 2 |
|  | 24c | Describe and explain any amendments to information provided at registration or in the protocol. | 2 |
| Support | 25 | Describe sources of financial or non-financial support for the review, and the role of the funders or sponsors in the review. | 20 |
| Competing interests | 26 | Declare any competing interests of review authors. | 20 |
| Availability of data, code and other materials | 27 | Report which of the following are publicly available and where they can be found: template data collection forms; data extracted from included studies; data used for all analyses; analytic code; any other materials used in the review. | 20 |

## Table S2. Search strategies.

| Number | Search strategy |
| --- | --- |
| 1 | (((((Lupus Erythematosus, Systemic[Title/Abstract]) OR (Systemic Lupus Erythematosus[Title/Abstract])) OR (Lupus Erythematosus Disseminatus[Title/Abstract])) OR (Libman-Sacks Disease[Title/Abstract])) OR (Disease, Libman-Sacks[Title/Abstract])) OR (Libman Sacks Disease[Title/Abstract]) |
| 2 | (((((((Lupus nephritis[Title/Abstract]) OR (Lupus Glomerulonephritis[Title/Abstract])) OR (Nephritis, Lupus[Title/Abstract])) OR (Lupus Nephritides[Title/Abstract])) OR (Nephritides, Lupus[Title/Abstract])) OR (Glomerulonephritis, Lupus[Title/Abstract])) OR (Glomerulonephritides, Lupus[Title/Abstract])) OR (Lupus Glomerulonephritides[Title/Abstract]) |
| 3 | ((((Phospholipids[Title/Abstract]) OR (Phosphatides[Title/Abstract])) OR (Phospholipid[Title/Abstract])) OR (antiphospholipid[Title/Abstract])) OR (anti-phospholipid[Title/Abstract]) |
| 4 | (((((Cardiolipins[Title/Abstract]) OR (Cardiolipin[Title/Abstract])) OR (Diphosphatidylglycerols[Title/Abstract])) OR (Diphosphatidylglycerol[Title/Abstract])) OR (Anticardiolipin[Title/Abstract])) OR (Anti-cardiolipin[Title/Abstract]) |
| 5 | (((((((((((((((beta 2-Glycoprotein I[Title/Abstract]) OR (beta 2 Glycoprotein I[Title/Abstract])) OR (Apo H[Title/Abstract])) OR (Endothelial Cell-Viability Maintaining Factor[Title/Abstract])) OR (Endothelial Cell Viability Maintaining Factor[Title/Abstract])) OR (beta(2)GPI[Title/Abstract])) OR (beta2-Glycoprotein I[Title/Abstract])) OR (beta2 Glycoprotein I[Title/Abstract])) OR (EC-VMFa[Title/Abstract]))) OR (Anticardiolipin Cofactor[Title/Abstract])) OR (Cofactor, Anticardiolipin[Title/Abstract])) OR (Apolipoprotein H[Title/Abstract])) OR (anti-β2 glycoprotein I[Title/Abstract])) OR (anti-β2 GPI[Title/Abstract])) OR (anti beta 2 GPI[Title/Abstract]) |
| 6 | ((((((((Lupus Coagulation Inhibitor[Title/Abstract]) OR (Lupus Anticoagulant Autoantibodies[Title/Abstract])) OR (Lupus Anticoagulant Autoantibody[Title/Abstract])) OR (Anticoagulant Autoantibody, Lupus[Title/Abstract])) OR (Autoantibody, Lupus Anticoagulant[Title/Abstract])) OR (Lupus Anticoagulant[Title/Abstract])) OR (Anticoagulant, Lupus[Title/Abstract])) OR (Coagulation Inhibitor, Lupus[Title/Abstract])) OR (Inhibitor, Lupus Coagulation[Title/Abstract]) |
| 7 | (((((((((Phosphatidylserines[Title/Abstract]) OR (Phosphatidyl Serines[Title/Abstract])) OR (Serines, Phosphatidyl[Title/Abstract])) OR (Serine Phosphoglycerides[Title/Abstract])) OR (Phosphoglycerides, Serine[Title/Abstract])) OR (Phosphatidylserine[Title/Abstract])) OR (Phosphatidyl Serine[Title/Abstract])) OR (Serine, Phosphatidyl[Title/Abstract])) OR (antiPhosphatidylserine[Title/Abstract])) OR (anti-Phosphatidylserine[Title/Abstract]) |
| 8 | (((((((((Prothrombin[Title/Abstract]) OR (Factor II[Title/Abstract])) OR (Blood Coagulation Factor II[Title/Abstract])) OR (Differentiation Reversal Factor[Title/Abstract])) OR (Factor, Differentiation Reversal[Title/Abstract])) OR (Coagulation Factor II[Title/Abstract])) OR (Factor II, Coagulation[Title/Abstract])) OR (II, Coagulation Factor[Title/Abstract])) OR (Anti-prothrombin[Title/Abstract])) OR (Antiprothrombin[Title/Abstract]) |
| 9 | ((Phosphatidylserine/prothrombin[Title/Abstract]) OR (Anti- Phosphatidylserine/prothrombin[Title/Abstract])) OR (antiPhosphatidylserine/prothrombin[Title/Abstract]) |
| 10 | ((((((((Phosphatidic Acids[Title/Abstract]) OR (Acids, Phosphatidic[Title/Abstract])) OR (Ammonium Phosphatidate[Title/Abstract])) OR (Phosphatidate, Ammonium[Title/Abstract])) OR (Phosphatidic Acid[Title/Abstract])) OR (Acid, Phosphatidic[Title/Abstract])) OR (Diacylglycerophosphates[Title/Abstract])) OR (anti-phosphatidic acid[Title/Abstract])) OR (antiphosphatidic acid[Title/Abstract]) |
| 11 | ((((((((((((((((((((Phosphatidylinositols[Title/Abstract]) OR (Inositol Phosphoglycerides[Title/Abstract])) OR (Phosphoglycerides, Inositol[Title/Abstract])) OR (Inositol Phospholipids[Title/Abstract])) OR (Phospholipids, Inositol[Title/Abstract])) OR (Phosphatidylinositol[Title/Abstract])) OR (Phosphoinositides[Title/Abstract])) OR (PtdIns[Title/Abstract])) OR (Inositide Phospholipids[Title/Abstract])) OR (Phospholipids, Inositide[Title/Abstract])) OR (Inositol Phospholipid[Title/Abstract])) OR (Phospholipid, Inositol[Title/Abstract])) OR (Inositide Phospholipid[Title/Abstract])) OR (Phospholipid, Inositide[Title/Abstract])) OR (Inositol Phosphoglyceride[Title/Abstract])) OR (Phosphoglyceride, Inositol[Title/Abstract])) OR (Phosphoinositide[Title/Abstract])) OR (Phosphatidyl Inositol[Title/Abstract])) OR (Inositol, Phosphatidyl[Title/Abstract])) OR (Anti-phosphatidylinositol[Title/Abstract])) OR (Antiphosphatidylinositol[Title/Abstract]) |
| 12 | (((((((((((Phosphatidylcholines[Title/Abstract]) OR (Phosphatidyl Cholines[Title/Abstract])) OR (Cholines, Phosphatidyl[Title/Abstract])) OR (Phosphatidyl Choline[Title/Abstract])) OR (Choline, Phosphatidyl[Title/Abstract])) OR (Choline Glycerophospholipids[Title/Abstract])) OR (Glycerophospholipids, Choline[Title/Abstract])) OR (Choline Phosphoglycerides[Title/Abstract])) OR (Phosphoglycerides, Choline[Title/Abstract])) OR (Phosphatidylcholine[Title/Abstract])) OR (Antiphosphatidylcholine[Title/Abstract])) OR (Anti-phosphatidylcholine[Title/Abstract]) |
| 13 | (((((((((Phosphatidylethanolamines[Title/Abstract]) OR (Cephalin[Title/Abstract])) OR (Ethanolamine Phosphoglyceride[Title/Abstract])) OR (Phosphoglyceride, Ethanolamine[Title/Abstract])) OR (Cephalins[Title/Abstract])) OR (Ethanolamine Phosphoglycerides[Title/Abstract])) OR (Phosphoglycerides, Ethanolamine[Title/Abstract])) OR (Ethanolamineglycerophospholipids[Title/Abstract])) OR (Antiphosphatidylethanolamine[Title/Abstract])) OR (Anti-phosphatidylethanolamine[Title/Abstract]) |
| 14 | ((protein C[Title/Abstract]) OR (Anti-protein C[Title/Abstract])) OR (Antiprotein C[Title/Abstract]) |
| 15 | ((((((((Protein S[Title/Abstract]) OR (Protein S, Vitamin K-Dependent[Title/Abstract])) OR (Protein S, Vitamin K Dependent[Title/Abstract])) OR (Vitamin K-Dependent Protein S[Title/Abstract])) OR (Vitamin K Dependent Protein S[Title/Abstract])) OR (Cofactor Protein S[Title/Abstract])) OR (Protein S, Cofactor[Title/Abstract])) OR (Anti-protein S[Title/Abstract])) OR (Antiprotein S[Title/Abstract]) |
| 16 | ((((((((((Annexin A2[Title/Abstract]) OR (Annexin II[Title/Abstract])) OR (Annexin II, P36[Title/Abstract])) OR (P36 Annexin II[Title/Abstract])) OR (Capactin I Heavy Chain[Title/Abstract])) OR (Lipocortin II[Title/Abstract])) OR (Annexin A2, P90 Complex[Title/Abstract])) OR (Annexin II, P90 Complex[Title/Abstract])) OR (Calpactin I[Title/Abstract])) OR (Anti-annexin A2[Title/Abstract])) OR (Antiannexin A2[Title/Abstract]) |
| 17 | (((((((((Annexin A5) OR (Anchorin CII)) OR (Annexin V)) OR (Calphobindin I)) OR (Endonexin II)) OR (Lipocortin V)) OR (Lipocortin-V)) OR (Placental Anticoagulant Protein I)) OR (Anti-membrane protein A5)) OR (Antimembrane protein A5) |
| 18 | 1 OR 2 |
| 19 | 3 OR 4 OR 5 OR 6 OR 7 OR 8 OR 9 OR 10 OR 11 OR 12 OR 13 OR 14 OR 15 OR 16 OR 17 |
| 20 | 19 AND 20 |

**Table S3.** Quality assessment of the case-control studies included in the meta-analysis using the NOS.

| Number | Source | Selection | | | | Comparability | Exposure | | |  |
| --- | --- | --- | --- | --- | --- | --- | --- | --- | --- | --- |
|  |  | Case definition | Cases representativeness | Controls selection | Contols definition | Comparability on the bias of design and analysis | Ascertainment of exposure | Same method of ascertainment | Non-response rate | Total |
| 1 | Helen I. Glueck et al. | 1 | 1 | 0 | 1 | 2 | 1 | 1 | 0 | 7 |
| 25 | Ana Barrera-Vargas et al. | 1 | 1 | 0 | 1 | 2 | 1 | 1 | 0 | 7 |
| 26 | Emanuel Farrugia et al. | 1 | 1 | 0 | 1 | 2 | 1 | 1 | 0 | 7 |
| 27 | Wafaa Gaber et al. | 1 | 1 | 0 | 1 | 2 | 1 | 1 | 0 | 7 |
| 44 | Geoffrey Frampton et al. | 1 | 1 | 0 | 1 | 1 | 1 | 1 | 0 | 6 |
| 47 | M H Houman et al. | 1 | 0 | 0 | 1 | 1 | 1 | 1 | 0 | 5 |
| 51 | P Alba et al. | 1 | 1 | 0 | 1 | 0 | 1 | 1 | 0 | 5 |
| 53 | Yuki Tsuruta et al. | 1 | 1 | 0 | 1 | 2 | 1 | 1 | 0 | 7 |
| 54 | Jing Luo et al. | 1 | 0 | 0 | 1 | 1 | 1 | 1 | 0 | 5 |
| 55 | Xuhui Zhong et al. | 1 | 1 | 0 | 1 | 1 | 1 | 1 | 0 | 6 |
| 57 | Miaochen Yu et al. | 1 | 1 | 0 | 1 | 0 | 1 | 1 | 0 | 5 |
| 58 | Pei Zhang et al. | 1 | 1 | 0 | 1 | 2 | 1 | 1 | 0 | 7 |
| 61 | Sulaiman M. Al-Mayouf et al. | 1 | 0 | 0 | 1 | 2 | 1 | 1 | 0 | 6 |
| 63 | Wanlin Cui et al. | 1 | 0 | 0 | 1 | 1 | 1 | 1 | 0 | 5 |
| 65 | WX Hu et al. | 1 | 0 | 0 | 1 | 1 | 1 | 1 | 0 | 5 |
| 66 | Joanna Kosałka-W˛egiel et al. | 1 | 1 | 0 | 1 | 1 | 1 | 1 | 0 | 6 |
| 67 | Eman M. Farid et al. | 1 | 0 | 0 | 1 | 2 | 1 | 1 | 0 | 6 |
| 70 | Claire Barber et al. | 1 | 0 | 0 | 1 | 2 | 1 | 1 | 0 | 6 |

## Table S4. Quality assessment of the cohort studies included in the meta-analysis using the NOS.

| Number | Source | Selection | | | | Comparability | Outcome | | |  |
| --- | --- | --- | --- | --- | --- | --- | --- | --- | --- | --- |
|  |  | Representativaness of exposed cohort | Selection of the non exposed cohort | Ascertainment of exposure | Demonstration that outcome was not present at start of study | Comparability on the bias of design and analysis | Assessment of outcome | Enough follw-up period | Adequacy of follow up | Total |
| 4 | Taraneh Mehrani et al. | 1 | 1 | 1 | 1 | 1 | 0 | 0 | 0 | 5 |
| 15 | Kotagal S. Kant et al. | 1 | 1 | 1 | 1 | 2 | 0 | 0 | 0 | 6 |
| 17 | Ruitong Gao et al. | 1 | 1 | 1 | 0 | 2 | 1 | 0 | 0 | 5 |
| 19 | Elena Gonzalo et al. | 1 | 1 | 1 | 1 | 0 | 1 | 0 | 0 | 5 |
| 20 | Natasha Jordan et al. | 1 | 1 | 1 | 1 | 1 | 1 | 1 | 0 | 7 |
| 21 | JM Mejıá-Vilet et al. | 1 | 1 | 1 | 1 | 1 | 1 | 1 | 0 | 7 |
| 22 | Gabriella Moroni et al. | 1 | 1 | 1 | 1 | 1 | 1 | 1 | 0 | 7 |
| 23 | R Shah et al. | 1 | 1 | 1 | 1 | 1 | 1 | 0 | 0 | 6 |
| 24 | Li-Hua Wu et al. | 1 | 1 | 1 | 1 | 1 | 1 | 1 | 0 | 7 |
| 28 | Indiran P. Naiker et al. | 1 | 1 | 1 | 1 | 2 | 1 | 0 | 0 | 7 |
| 32 | Marıá Galindo et al. | 1 | 1 | 1 | 1 | 1 | 0 | 1 | 1 | 7 |
| 35 | Y. Shen et al. | 1 | 1 | 1 | 1 | 2 | 1 | 1 | 0 | 7 |
| 36 | R Silvariño et al. | 1 | 1 | 1 | 1 | 2 | 1 | 1 | 0 | 8 |
| 41 | Hui Zheng et al. | 1 | 1 | 1 | 1 | 2 | 1 | 0 | 0 | 7 |
| 45 | M Abu-Shakra et al. | 1 | 1 | 1 | 1 | 1 | 1 | 0 | 0 | 6 |
| 46 | Eric Descombes et al. | 1 | 1 | 1 | 1 | 1 | 1 | 1 | 0 | 7 |
| 50 | E. Descloux et al. | 1 | 1 | 1 | 1 | 1 | 1 | 1 | 1 | 8 |
| 56 | Savino Sciascia et al. | 1 | 1 | 1 | 1 | 1 | 1 | 1 | 1 | 8 |
| 59 | Bianka Perge et al. | 1 | 1 | 1 | 1 | 1 | 1 | 1 | 0 | 7 |
| 60 | Sulaiman M. Al-Mayouf et al. | 1 | 1 | 1 | 1 | 1 | 1 | 0 | 0 | 6 |
| 62 | Seoung Wan Nam et al. | 1 | 1 | 1 | 1 | 1 | 1 | 1 | 0 | 7 |
| 64 | J. FONT et al. | 1 | 1 | 1 | 1 | 1 | 1 | 1 | 1 | 8 |

**Table S5.** Quality assessment of the cross-sectional studies included in the meta-analysis using the NOS.

| Number | Source | Study sample selection | | Assessment of exposure and outcome | | Confounding factor | | Total | |
| --- | --- | --- | --- | --- | --- | --- | --- | --- | --- |
|  |  | Representativeness  of the study sample | Sample size | Assessment of the  exposure | Assessment of the  outcome | Adjustment for  confounder | Assessment of confounder |  |  |
| 2 | K. E. Moss et al. | 1 | 0 | 2 | 2 | 1 | 1 | 7 |  |
| 3 | P. Stratta et al. | 1 | 0 | 2 | 2 | 1 | 1 | 7 |  |
| 5 | Ioannis Parodis et al. | 1 | 0 | 2 | 2 | 1 | 1 | 7 |  |
| 6 | Leyre Riancho-Zarrabeitia et al. | 1 | 0 | 2 | 2 | 2 | 1 | 8 |  |
| 7 | Savino Sciascia et al. | 1 | 0 | 2 | 2 | 1 | 0 | 6 |  |
| 8 | Małgorzata Wisłowska et al. | 1 | 0 | 2 | 2 | 0 | 1 | 6 |  |
| 9 | Chindarat Natejumnong et al. | 1 | 0 | 2 | 2 | 0 | 1 | 6 |  |
| 10 | S.Loizou et al. et al. | 1 | 0 | 2 | 2 | 0 | 0 | 5 |  |
| 11 | Juan Manuel Anaya et al. | 1 | 0 | 2 | 2 | 0 | 1 | 6 |  |
| 12 | Maria G. Tektonidou et al. | 1 | 0 | 2 | 2 | 0 | 1 | 6 |  |
| 13 | Boonyarit Cheunsuchon et al. | 1 | 0 | 2 | 2 | 1 | 0 | 6 |  |
| 14 | Eric Daugas et al. | 1 | 0 | 2 | 2 | 0 | 0 | 5 |  |
| 16 | Juan M. Miranda et al. | 1 | 0 | 2 | 2 | 1 | 0 | 6 |  |
| 18 | Jakob Gerhardsson et al. | 1 | 0 | 2 | 2 | 1 | 0 | 6 |  |
| 29 | S. Bhandari et al. | 1 | 0 | 2 | 2 | 1 | 0 | 6 |  |
| 30 | Danielle Cohen et al. | 1 | 0 | 2 | 2 | 0 | 0 | 5 |  |
| 31 | Gian Luca Erre et al. | 1 | 0 | 2 | 2 | 1 | 0 | 6 |  |
| 33 | Juan M. Miranda et al. | 1 | 0 | 2 | 2 | 1 | 0 | 6 |  |
| 34 | M. Perdiguero et al. | 1 | 0 | 2 | 2 | 1 | 0 | 6 |  |
| 37 | Di Song et al. | 1 | 0 | 2 | 2 | 1 | 0 | 6 |  |
| 38 | G. Hernández-Molina et al. | 1 | 0 | 2 | 2 | 2 | 1 | 8 |  |
| 39 | Faisal Naseeb et al. | 1 | 0 | 2 | 2 | 1 | 0 | 6 |  |
| 40 | Jianna Zhang et al. | 1 | 0 | 2 | 2 | 1 | 0 | 6 |  |
| 42 | Yan Zhou et al. | 1 | 0 | 2 | 2 | 1 | 0 | 6 |  |
| 43 | D. P. D'CRUZ et al. | 1 | 0 | 2 | 2 | 0 | 0 | 5 |  |
| 48 | Gözde Sevgi Kart Bayram et al. | 1 | 0 | 2 | 2 | 1 | 1 | 6 |  |
| 49 | Gudrun E Norby et al. | 1 | 0 | 2 | 2 | 1 | 0 | 6 |  |
| 52 | D-C Varela et al. | 1 | 0 | 2 | 2 | 1 | 0 | 6 |  |
| 69 | A. Šipek-Dolničan et al. | 1 | 0 | 2 | 2 | 0 | 0 | 5 |  |


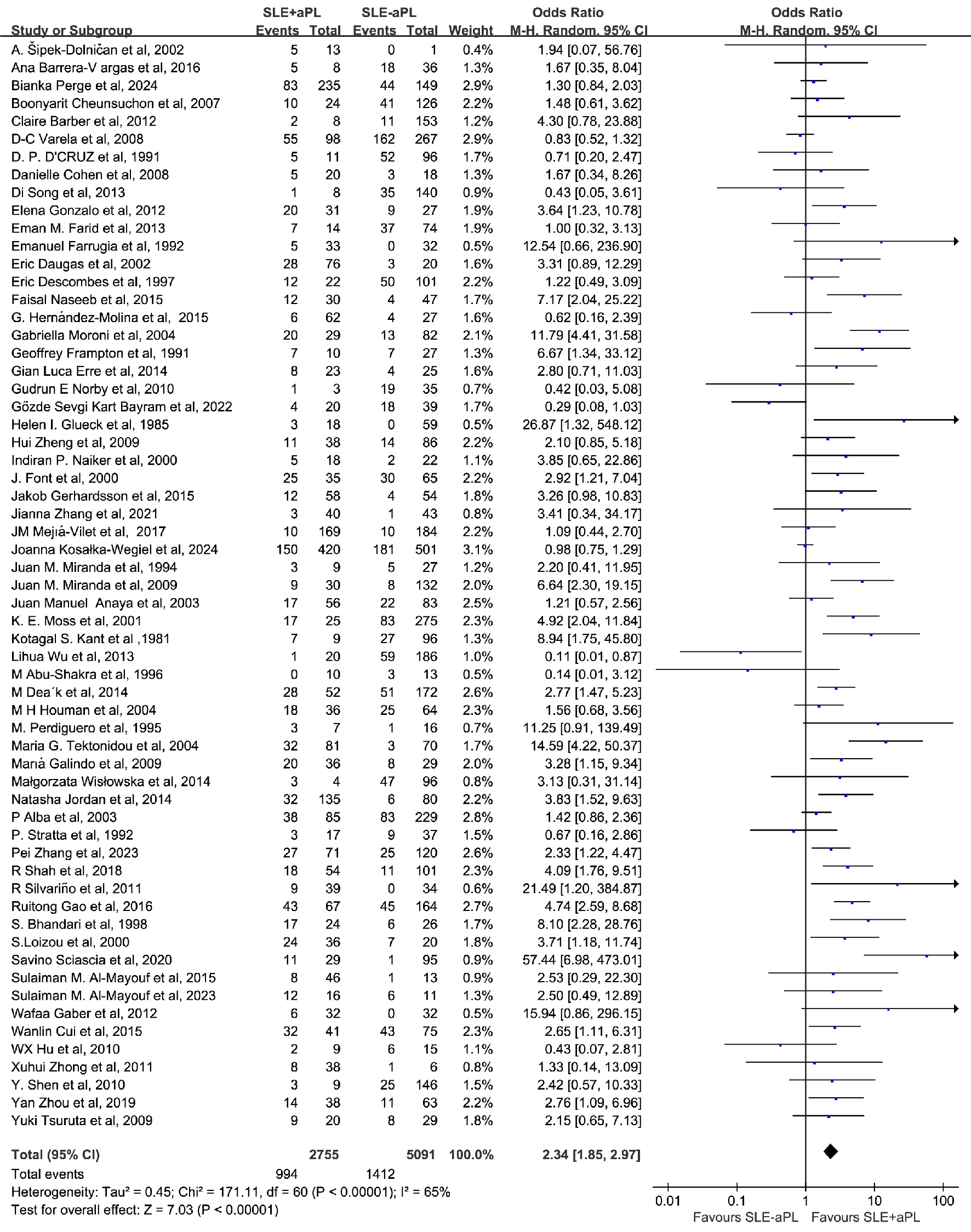


**Figure S1.** Association between overall aPL and biopsy-proved renal injury in SLE.


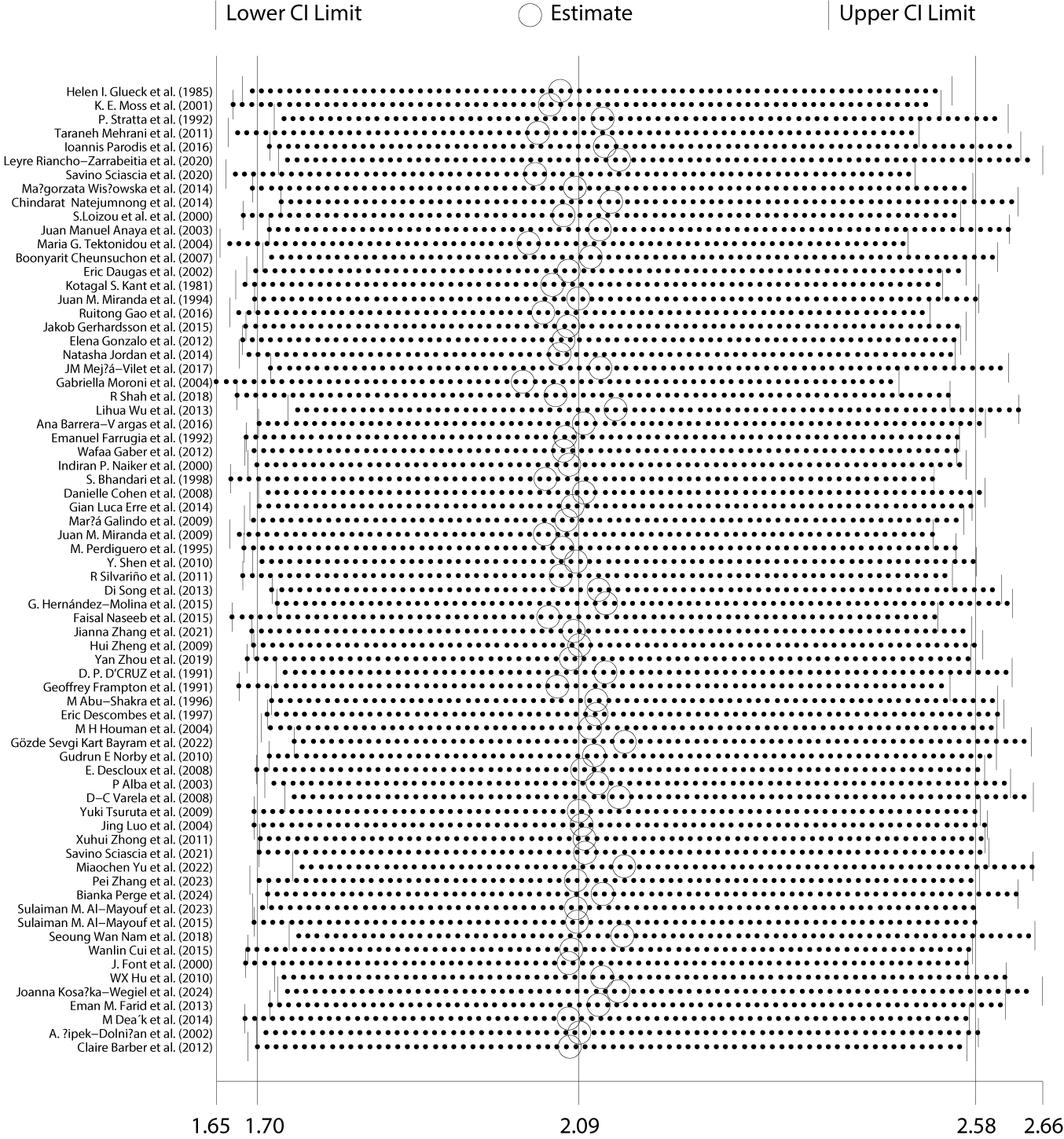


## Figure S2. Sensitivity analysis of the included studies.


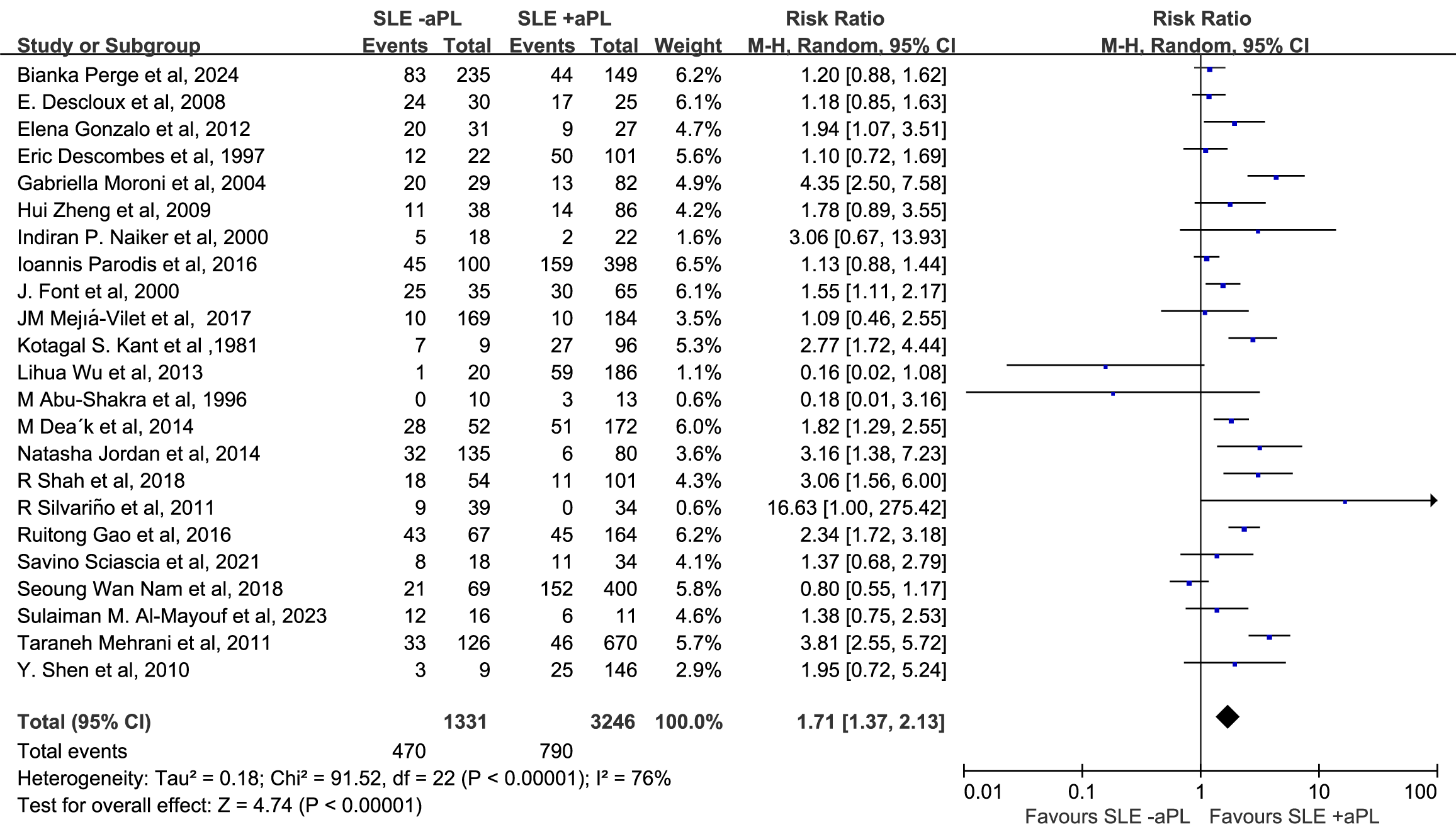


**Figure S3.** Risk ratio between aPL positivity and renal injury for cohort studies.


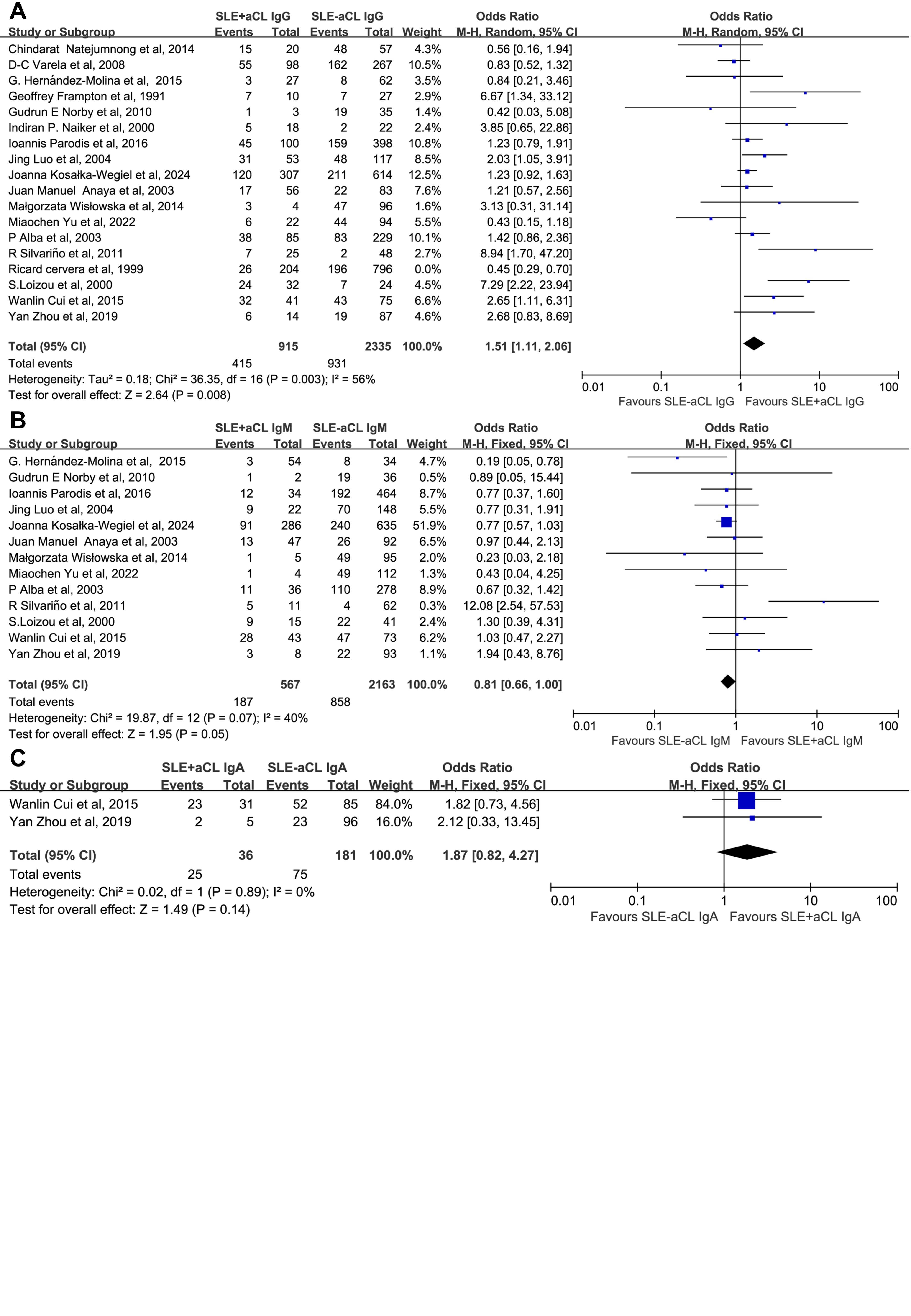


**Figure S4.** Association between IgG, IgM, and IgA isotypes of aCL antibodies and renal injury: (A) aCL IgG, (B) aCL IgM, (C) aCL IgA.


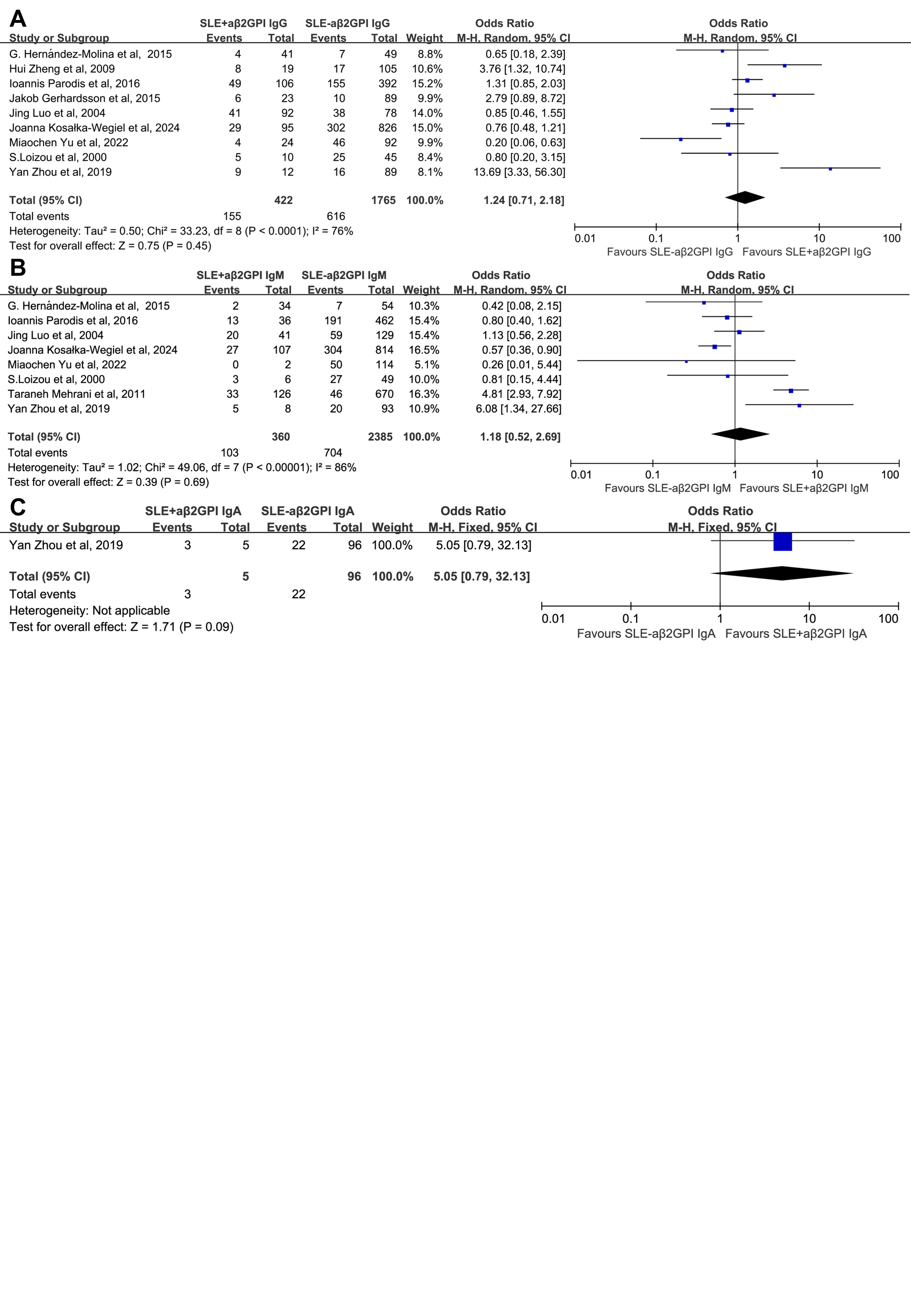


**Figure S5.** Association between aβ₂GPI antibodies and renal injury: (A) aβ₂GPI IgG isotype, (B) aβ₂GPI IgM isotype, (C) aβ₂GPI IgA isotype.

**
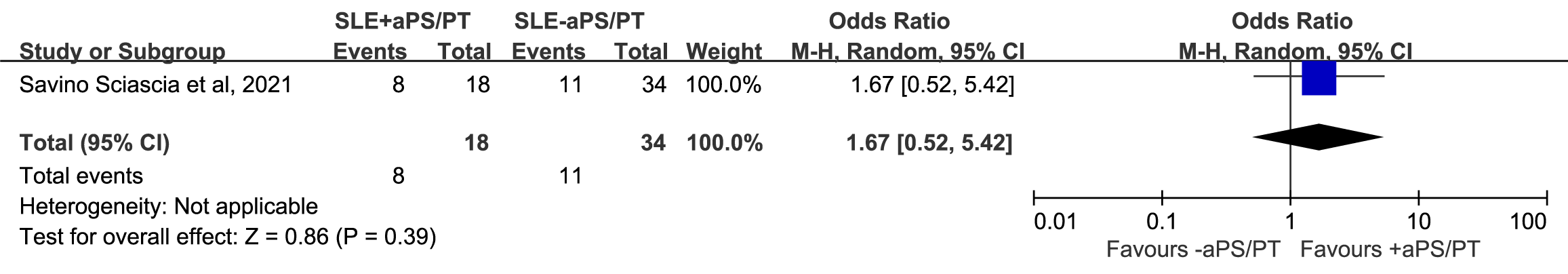
**

**Figure S6.** Association between aPS/PT antibodies and renal injury.


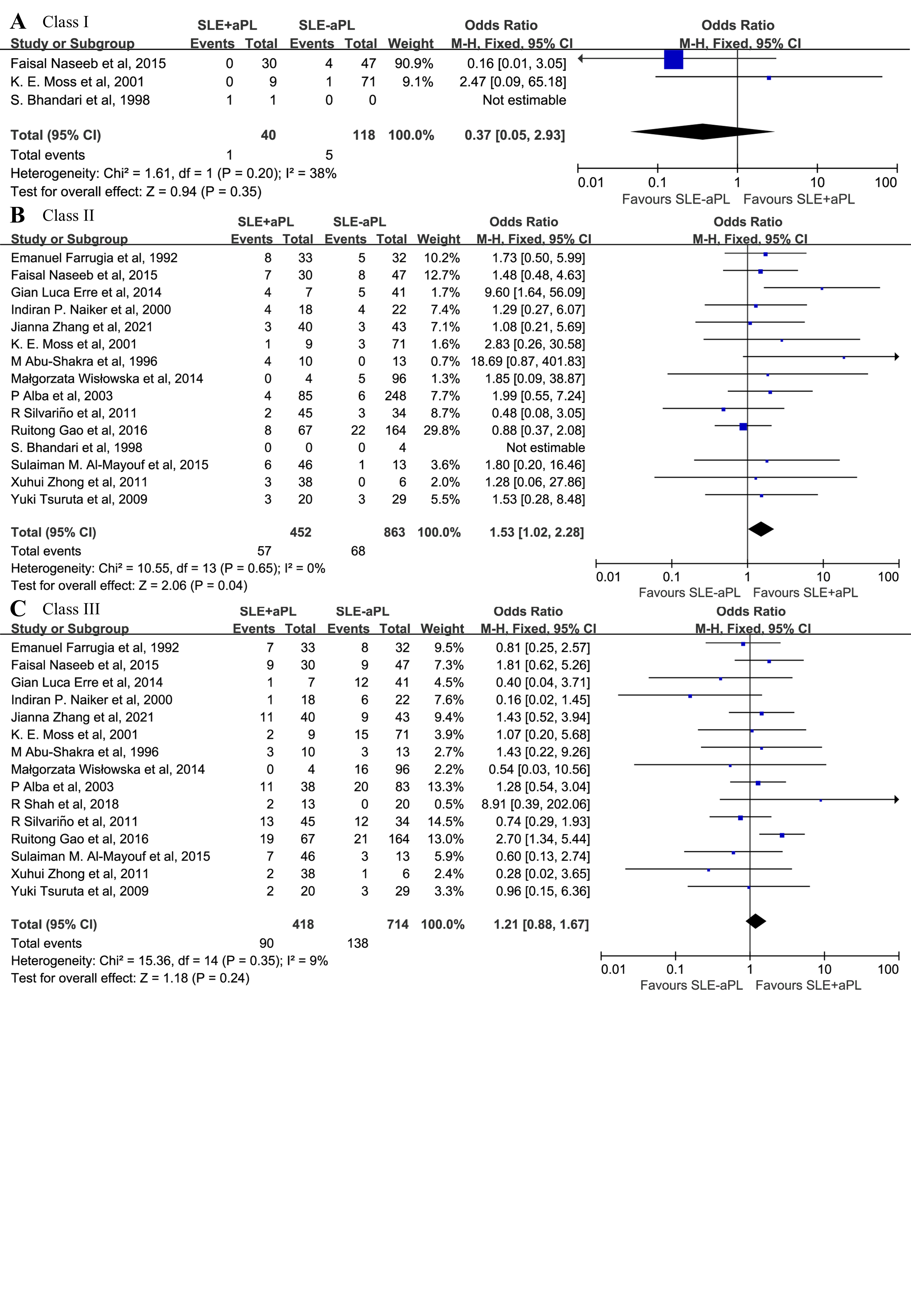


**Figure S7.** Association between aPL and histopathological classification of lupus nephritis: (A) class I, (B) class II, (C) class III.


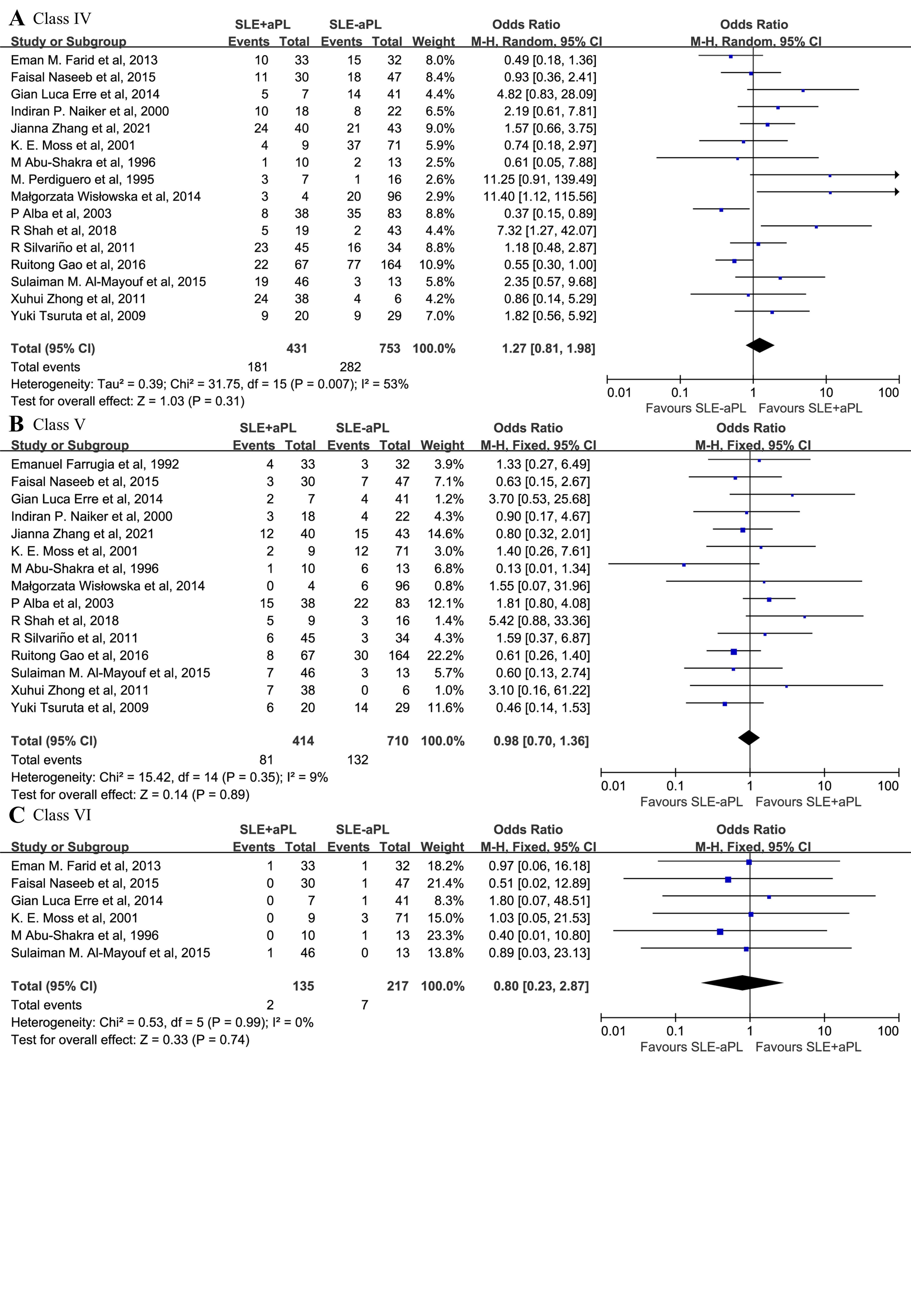


**Figure S8.** Association between aPL and histopathological classification of lupus nephritis: (A) class IV, (B) class V, (C) class VI.
